# Engineering Extended Release Profiles for Biologic Formulations via Chemical Cross-Linking of Poloxamer 407 Hydrogels

**DOI:** 10.1021/acsomega.5c02571

**Published:** 2025-11-24

**Authors:** Jungsoo Park, Yu-Jiun Lin, Kingshuk Dutta, Seth Forster, Grace Okoh, Yu Tian, Yingkai Liang

**Affiliations:** 1 Discovery Pharmaceutical Sciences, 2793 Merck & Co., Inc., West Point, Pennsylvania 19486, United States; 2 Materials and Biophysical Characterization, 2793 Merck & Co., Inc., Rahway, New Jersey 07065, United States

## Abstract

Hydrogels, networks of hydrophilic polymers known for their water retention capacity, biodegradability, and biocompatibility, are ideal for the sustained and extended delivery of biologics. Because in situ hydrogels can form at the administration site in response to external stimuli, they can provide noninvasive and localized delivery of biotherapeutics. In particular, poloxamer 407 (P407), an “A–B–A” triblock copolymer, composed of hydrophilic poly­(ethylene oxide) (block A) and hydrophobic poly­(propylene oxide) (block B), exhibits reversible thermal property: liquid at room temperature and gelling at elevated temperatures. This characteristic, combined with its low toxicity and excellent chemical compatibility, makes P407 an attractive polymer for drug delivery applications. However, its low mechanical strength and weak gel stability have limited its broader use in therapeutic applications. To address this challenge, chemically cross-linked P407 hydrogels were developed using acrylate-modified P407 and thiol-terminated eight-arm polyethylene glycol with two different molecular weights (MWs) via Michael-type addition. Chemical cross-linking enhanced the mechanical strength of P407 hydrogels, enabling extended in vitro release of bovine serum albumin (BSA), human plasma immunoglobulin G antibody (IgG), and adalimumab for up to 70 days with tunable release kinetics profile. Biophysical and functional characterization via circular dichroism, size exclusion chromatography, capillary electrophoresis-sodium dodecyl sulfate, and enzyme-linked immunosorbent assay indicated that the hydrogels did not adversely affect the structural conformation, stability, and in vitro potency of the encapsulated biologics. This study highlights improved P407 hydrogel stability and tunable release profiles by incorporating acrylate reactive cross-linkers with two different MWs, providing insights for the application of sustained and controlled release of biologic.

## Graphical abstract

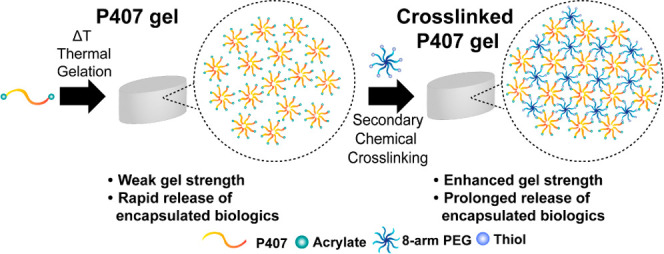

## 1.Introduction

Biologics, such as antibodies, offer significant therapeutic advantages due to their target specificity and improved pharmacokinetic profiles.^1^ However, these large molecules often face various challenges in delivery due to the delicate nature of their structures, including aggregation, fragmentation, chemical modification, and protein denaturation, which can decrease their bioactivity and potentially induce immunogenicity.^2–5^ Moreover, due to the limited permeability across biological barriers, biologics typically require parenteral administration and regular dosing, potentially impacting the patient compliance and outcome.^2,6^ To address the challenges in biologics delivery, hydrogels, a three-dimensional molecular network of hydrophilic polymers with great water retention capacity as well as biodegradability and biocompatibility, have been extensively utilized.^7–10^ Their porous structure and tunable properties create a protective environment for biologics, facilitating controlled release and maintaining constant drug concentration over time, thereby improving biologics’ stability, reducing administration frequency and side effects.^9,11,12^

In situ hydrogels are promising for biologic delivery, being able to transition from injectable solutions to gels at the administration site through various stimuli.^13–15^ Unlike preformed hydrogels requiring surgical implantation, in situ hydrogels offer minimally invasive delivery option. In particular, temperature-sensitive in situ hydrogels have attracted considerable interest,^16^ since the gelation can be conveniently triggered at physiological temperature (37 °C) within minutes to hours, depending on the formulation, avoiding potential degradation from other stimuli mechanisms such as pH, UV, ionic strength, or enzyme-induced gelation.^13–16^ Among polymers used for these temperature-sensitive in situ gels, poloxamer 407 (P407) has been commonly used due to its low toxicity and compatibility with numerous chemicals as an excipient, is certified as a Generally Recognized as Safe (GRAS) by U.S. Food and Drug administration (FDA),^17,18^ and is supported by the literature indicating that P407 hydrogels do not exert in vitro cytotoxicity^19,20^ and do not cause significant inflammation or adverse effects in vivo*.* ^21–23^

P407 is a “A–B–A” triblock copolymer with a molecular weight of 12,500 Da composed of 101 repeat units of poly­(ethylene oxide) (PEO) as the hydrophilic block A and 56 repeat units of poly­(propylene oxide) (PPO) as the hydrophobic block B. 15–30 weight percent (wt %) P407 aqueous solution can form gel at body temperature (37 °C).^17^ The mechanism underlying this transition involves elevated temperature disrupting the hydration layer around P407 molecules, promoting hydrophobic interactions among the PPO blocks and triggering micelle formation, which leads to gelation.^17,24^ This temperature-dependent thermogelling property has been utilized to formulate gels to deliver biologics such as recombinant human growth hormone,^25^ insulin,^26^ and cytokines.^22,27^ However, P407’s short in situ residence time (up to few days) and low mechanical strengths limit its drug delivery capabilities.^17,18^ To address these issues, attempts have been made to extend the release by cross-linking P407 via photopolymerization.^28–30^ However, photopolymerization poses inherent risks to biologics, as the reaction itself is an exothermic reaction, photoinitiating condition such as UV light can induce protein degradation or denaturation.^31^

Advancements in hydrogel technology in recent decades have highlighted the potential of spontaneous/orthogonal chemical cross-linking, particularly through Michael-type addition, as an effective approach to enhance physical/thermal hydrogel stability for tissue engineering and controlled release applications.^32,33^ For instance, Hubbell et al. developed a cross-linked poloxamer 407 (P407) gel using hexathiol and diacrylate modifications, resulting in improved gel stability and mechanical strength suitable for cell encapsulation.^34,35^ Similarly, functionalized P407 copolymers using esterification reaction have been employed to develop hydrogels with enhanced stability for quantifiable release of methylene blue as well as 3T3 fibroblast encapsulation.^36,37^ Additionally, maleimide and furyl group-functionalized poloxamine (Tetronic 1107) have been employed to form a hydrogel via Diels–Alder reaction, which allowed for tunable release profiles of bevacizumab over 115 days while maintaining significant potency.^38^

Building on these innovative approaches, this work aims to strategically integrate the in situ forming properties of thermoresponsive P407 with physiologically relevant Michael-type addition cross-linking chemistry for enhanced gel stability and extended release of encapsulated biologics. The rapid gelation of P407 at body temperature enables easy injectability, while the subsequent Michael-type reactions gradually cure and stabilize the hydrogel, increasing its resistance to dissolution for sustained delivery. Chemical cross-linking of P407 hydrogels was performed using P407 diacrylate and thiol-terminated 8-arm polyethylene glycol (PEG) of different molecular weights (MWs). It was hypothesized that the P407 hydrogel network could be covalently reinforced by incorporating PEG linkers of different MWs, thus preventing rapid gel degradation and allowing for the modulation of gel strength and the tuning of release profiles, while achieving the in situ gel formation upon injection. This study focuses on assessing the physical properties of chemically cross-linked P407 hydrogels, including swelling ratios and rheological characteristics, followed by the encapsulation of various biologics including bovine serum albumin (BSA), human serum immunoglobulin (IgG), and a therapeutic monoclonal antibody (mAb) adalimumab. The influence of the molecular weight (MW) of the PEG cross-linker on the release rates of these biologics was evaluated. Moreover, the biophysical integrity of these released cargoes was thoroughly assessed through size exclusion chromatography (SEC), capillary electrophoresis-sodium dodecyl sulfate (CE-SDS), and circular dichroism (CD). Finally, the biological functionality of the adalimumab released from the chemically cross-linked P407 hydrogel was confirmed by enzyme-linked immunosorbent assay (ELISA). The findings from this study offer valuable insights for designing long-acting hydrogel formulations that could serve as a simple yet elegant approach for sustained therapeutic delivery, paving the way for enhanced patient outcomes in biopharmaceutical applications.

## 2 Results

### 2.1 Chemically Cross-Linked P407 Hydrogels and Their Rheological Properties

Chemically cross-linked P407 hydrogels were formed using a 25-weight percent (wt %) solution of P407-diacrylate polymer and 8-arm-PEG-SH cross-linkers (MW: 10 or 20 kDa) via Michael-type addition in 1× PBS (pH 7.4) at a 1:1 stoichiometric ratio of thiol to acrylate groups (Scheme 1). This method was chosen for its rapid reaction kinetics and favorable reaction conditions under biologics-friendly, physiological pH.^39^ In this study, 25 wt % P407-diacrylate polymer solution was chosen to formulate chemically cross-linked hydrogels based on the appropriate gelation time (ca. 5 min) as previously reported.^19^ This balance ensures sufficient gel strength while allowing adequate handling time and minimizing the risk of needle clogging during injection. An 8-arm-PEG-SH was selected instead of a 4-arm-PEG-SH because a higher arm number increases cross-linking density, reduces network mesh size, and enables more prolonged release of encapsulated cargoes.^40–43^ As shown in Figure 1a, the P407 hydrogel without a PEG cross-linker (henceforth termed P407 gel) exhibits reversible temperature-dependent sol–gel transition, remaining as a solution at 4 °C and forming a gel at 37 °C. In contrast, P407-based hydrogels fully chemically cross-linked with 8-arm PEG10K-SH or 8-arm PEG20K-SH (henceforth termed P407-10K gel and P407-20K gel, respectively) remain intact regardless of temperature, visually indicating hydrogel stabilization. To determine the cross-linking efficiency, hydrogels were disintegrated with 1× PBS (pH 10.0) at 37 °C for 3 days and then buffer exchanged back to 1× PBS (pH 7.4) using Amicon 10K molecular weight cutoff (MWCO) spin filters. It is well-known that hydrolysis of propylene oxide (the hydrophobic block of P407), can be accelerated in alkaline conditions. Hydrolysis would disrupt the hydrophobic interactions that stabilize the micelles within the gel matrix, compromising the structural integrity of the hydrogel, leading to accelerated disintegration. Furthermore, a previous report indicated that P407 gel (26 wt %) took 7 days to completely disintegrate at 37 °C.^44^ Therefore, pH 10.0, 1× PBS buffer was used to expedite the disintegration of the P407-10K gel and the P407-20K gel. The unreacted 8-arm-PEG-SH linkers were collected in filtrate and quantified by Ellman’s reagent,^45^ which reacts effectively with sulfhydryl groups in a pH range of approximately 7.0 to 9.0. The results showed that free thiol was detected at a very low level (193.4 μg and 209.2 μg of 8-arm-PEG10K-SH and 8-arm-PEG20K-SH detected, respectively), indicating that approximately 99% of the 8-arm-PEG-SH had reacted with the P407-diacrylate polymer to form cross-links (Figure S1). The viscoelastic properties of the P407-10K gel and P407-20K gel were then evaluated further by oscillatory rheology (Figure 1b–e). The storage modulus (*G*′) (indicative of gel-like behavior) and the loss modulus (*G*″) (indicative of liquid-like behavior) were measured over an angular frequency range of 0.1–10 rad/s. At 37 °C, the P407 gel, P407-10K gel, and P407-20K gel exhibited *G*′ > *G*″, confirming a gel-like behavior (Figure 1b). The P407-10K gel and P407-20K gel exhibited *G*′ values up to 2.1-fold higher values than P407, indicating improvement in gel strength due to chemical cross-linking. The P407-10K gels had *G*′ values up to 1.2-fold higher than P407-20K gels across the angular frequency range, owing to the molecular weight difference of the 8-arm-PEG-SH cross-linkers. Lower molecular weight PEG cross-linkers would form a denser, tightly cross-linked network, while high molecular weight cross-linkers would create a looser network with larger cavities.^46,47^ At 4 °C, P407 displayed liquid-like behavior (*G*″ > *G*′), while P407-10K and P407-20K gels maintained a gel-like behavior (*G*′ > *G*″) throughout the angular frequency range (Figure 1b). The reduced *G*′ values at 4 °C for the P407-10K gel and P407-20K gel suggest a transition from spherical micelles to linear triblock copolymer structures, yet the integrity was preserved due to chemical cross-linking.

**Scheme 1:**
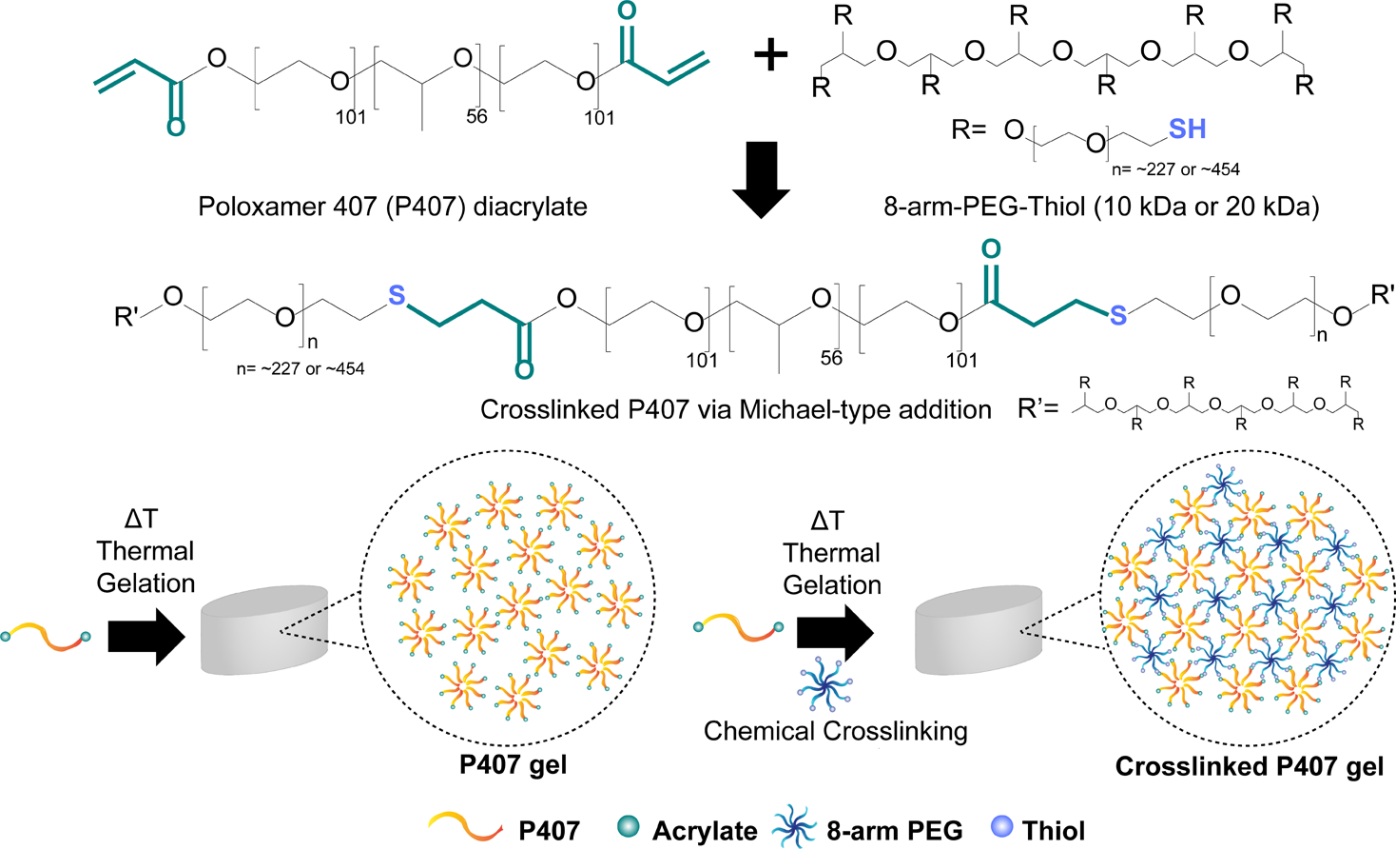
(a) Structure of Poloxamer 407 (P407) Diacrylate and 8-arm-PEG-thiol Used in This Study;^a^ (b) Schematic Describing the Formation of Physical P407 Hydrogel at Elevated Temperature and Chemically Cross-Linked P407 Hydrogel via Michael-Type Addition at Elevated Temperatures

a P407-diacrylate polymer can be cross-linked with 8-arm-PEG-thiol via Michael addition.

**Figure 1:**
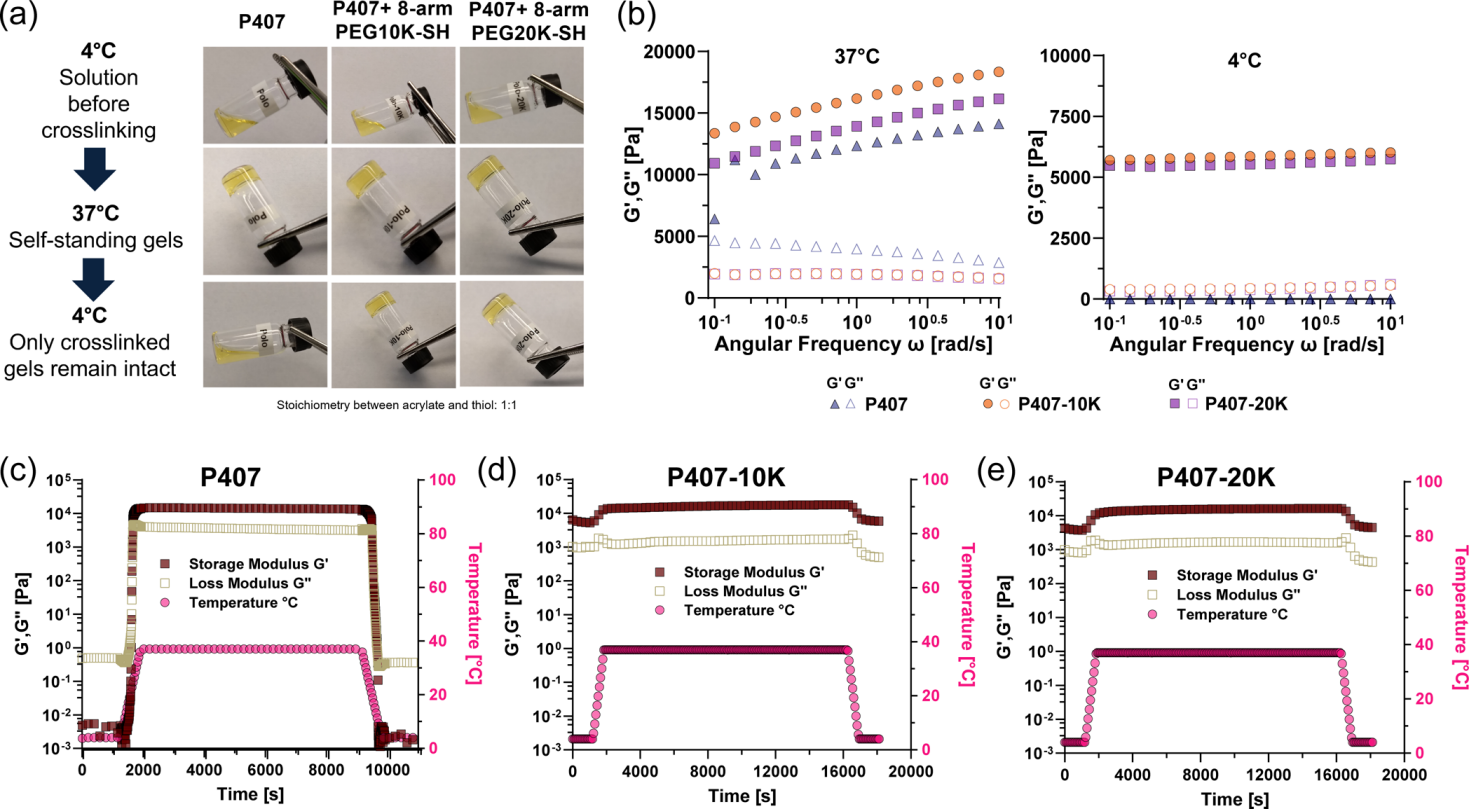
(a) 25 wt % P407 gel without a PEG cross-linker (P407 gel) exhibits temperature-dependent reversible gelation property where it remains in a solution state at 4 °C and forms a hydrogel at 37 °C. Chemically PEG cross-linked P407 hydrogels, however, remain at a gel state regardless of temperature. (b) Rheological characterization of P407 hydrogel formulations at 37 °C and 4 °C using frequency-dependent oscillatory shear sweep with a fixed strain of 3%. Rheological behavior of (c) P407 gel, (d) P407-10K (P407-diacrylate polymer chemically cross-linked with 8-arm-PEG10K-SH) gel, and (e) P407-20K (P407-diacrylate polymer chemically cross-linked with 8-arm-PEG20K-SH) gel as a function of time via temperature sweep at 6 rad/s and 3% strain.

*G*′ and *G*″ values of the P407 gel, P407-10K gel, and P407-20K gel were also analyzed as a function of time and temperature by performing oscillatory time sweep experiment. Time sweep experiments indicated that P407 transitioned to a gel state at approximately 23 °C, with *G*′ rapidly increasing to reach a plateau at 37 °C but exhibiting reversible sol–gel transition (*G*″ > *G*′) when the temperature was returned to 4 °C (Figure 1c). The gelation kinetics of the P407-10K gel and P407-20K gel were also determined, showing that gelation occurred relatively slow compared to the P407 gel, which completed gelation within 30 min after the temperature was increased from 4 °C to 37 °C (Figure S2). The gradual increase of storage modulus (*G*′) without reaching a plateau indicated full chemical cross-linking had not yet been achieved. Once fully cross-linked, the P407-10K gel and P407-20K gel maintained *G*′ > *G*″ throughout the temperature range (Figure 1d,e), confirming enhanced gel stability and network reinforcement due to chemical cross-linking as depicted in Figure 1a.

### 2.2 Swelling Properties of P407-10K Gels and P407-20K Gels

The swelling ratios of the P407-10K and P407-20K gels were then quantified, as this property is crucial for hydrogels intended for biomedical and pharmaceutical applications. The swelling ratio inversely correlates with cross-linking density, influencing the diffusion rate and release kinetics of encapsulated biologics, greater swelling leading to faster release of biologics.^46^ It was hypothesized that P407-10K gels would have a lower swelling ratio than P407-20K gels due to their shorter PEG length, resulting in higher cross-linking density and reduced water absorption (Figure 2a). As anticipated, the equilibrium swelling ratios of P407-20K gels were approximately 1.3-fold higher than those of P407-10K gels over a 70 day release period (Figure 2b). The repeated measure analysis revealed that P407-10K gels and P407-20K gels indeed have statistically different swelling profiles, except for the period between days 14 and 35 (Table S1). We hypothesize that this is due to the hydrogels reaching the equilibrium swelling state. However, after day 35, hydrogel degradation may occur, leading to statistically significant differences in swelling ratios once again. Similar findings by Hubbell and Metters also indicated that swelling ratios increase with the increased molecular weight of cross-linkers at later time points.^43^ These results demonstrate that the network density and swelling of the cross-linked P407-based hydrogels can be tuned by varying the molecular weight of cross-linkers, offering insights for tailoring P407-based hydrogel formulations to achieve various drug release profiles for biomedical and pharmaceutic applications.

**Figure 2:**
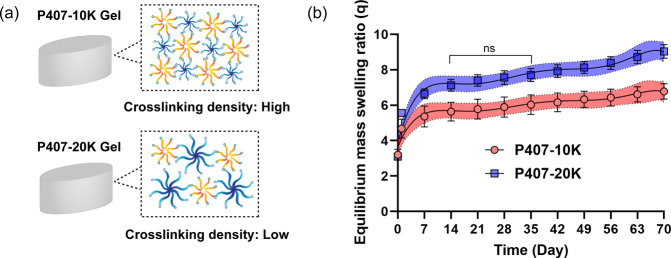
(a) Due to the shorter arm length of the 8-arm-PEG-SH cross-linker used, the cross-linking density is expected to be higher in P407-10K gel compared to P407-20K gel. (b) Equilibrium mass swelling ratio of P407-10K gel and P407-20K gel in 1× PBS, pH 7.4 at 37 °C (average ± S.D, *n* = 3) with 95% confidence band. Repeated measures analysis revealed that the swelling ratio between the P407-10K gel and P407-20K gel is statistically significantly different from one another except for day 14 to day 35 (*p* < 0.05).

### 2.3 Release Kinetics of BSA from P407-10K Gels and P407-20K Gels

A key application of in situ hydrogels in drug delivery is their ability to extend the release of biologics over a prolonged period. Hence, in this study, BSA (40 mg/mL, based on the manufacturer’s solubility test) was used as a model protein to evaluate the release kinetics of BSA encapsulated in P407-10K gels and P407-20K gels (the encapsulation and release details are described in the method section). The release of BSA from cross-linked hydrogel was measured every 24 h for the first 4 days and then every week until the gels completely disintegrated due to matrix degradation. For the P407 gels, 100% cumulative release of BSA was achieved within 24 h, exhibiting nearly zero order release (Figure 3a), consistent with literature P407 hydrogel degrades easily in aqueous solutions, leading to rapid release of encapsulated cargoes within 1 or 2 days via surface erosion.^13,48,49^ In contrast, P407-10K gels and P407-20K gels exhibited a sustained BSA release profile lasting up to 42 days with cumulative release continuing over a prolonged period of 70 days (Figure 3b). Notably, no significant burst release was observed. This can be attributed to the hydrogel mesh sizes (distance between two entanglement points, ξ), which can be calculated from the storage modulus *G*′ based on rubber elasticity theory.^50^ The mesh sizes of P407-10K gels and P407-20K gels at 37 °C are approximately 6.2–6.8 and 6.4–7.3 nm, respectively (Table S2). Given that the hydrodynamic diameter of BSA is reported to be 7.0 nm,^51^ the mesh size of the hydrogel would limit the rapid diffusion of BSA at initial time points. Moreover, compared to P407-20K gels, P407-10K gels exhibited a slower cumulative BSA release up to the 42 day sustained release phase (P407-10K gels: 16.6 ± 0.1%, and P407-20K gels: 22.6 ± 0.1%). This difference was statistically significant based on repeated measures analysis (except at day 42, Table S3), which can be attributed to the smaller MW of the cross-linker in P407-10K resulting in increased cross-linking density and smaller swelling ratio consistent with the Flory–Rehner theory^52^ as well as the reported literature.^43,47^

**Figure 3:**
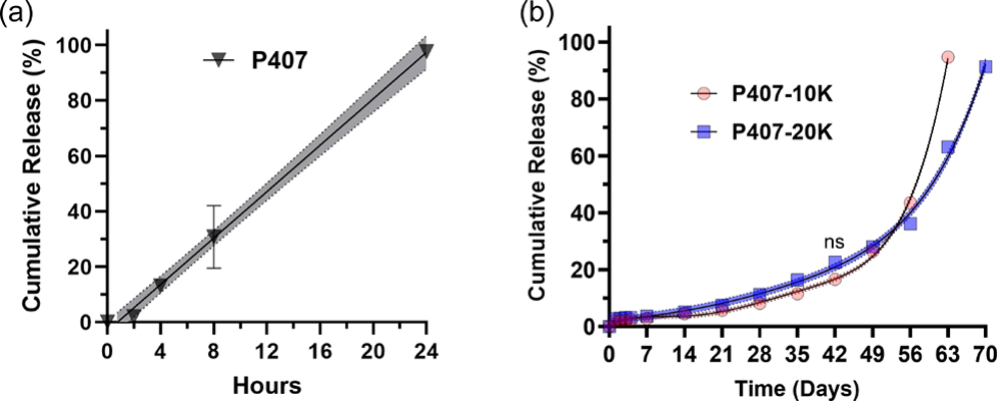
Cumulative release of bovine serum albumin (BSA) from (a) P407 and (b) P407-10K and P407-20K gel formulations in 1× PBS, pH 7.4 at 37 °C (average ± S.D, *n* = 3), with 95% confidence band. Repeated measure analysis revealed that the cumulative release percentage of BSA between the P407-10K gel and P407-20K gel is statistically significantly different from one another except for day 42 (*p* < 0.05).

The cumulative release of BSA was noted to be less than 22% for both P407-10K gels and P407-20K gels up to day 42. According to Hirayama et al. and Lee et al., BSA contains one free sulfhydryl group that can contribute to its retention in the hydrogel through chemical conjugation,^53,54^ suggesting a Michael-type addition between BSA and the P407-diacrylate polymer that could slow BSA release. To verify this hypothesis, BSA-encapsulated P407-10K gels and P407-20K gels were formulated and disintegrated, and BSA was extracted via precipitation. Ellman’s reagent^45^ then was used to quantify the concentration of free sulfhydryl groups in the BSA extracted from P407-10K gels and P407-20K gels (6 mg/mL, 90.4 μM), based on a standard curve generated using known concentrations of freshly prepared BSA solution in 1× PBS (pH 7.4). Compared to the free sulfhydryl group concentration of freshly prepared 6 mg/mL BSA solution (89.9 ± 1.1 μM), 6 mg/mL BSA solution extracted from P407-10K gels (concentration of free sulfhydryl group: 58.7 ± 4.9 μM) and P407-20K gels (concentration of free sulfhydryl group: 30.3 ± 5.3 μM) showed approximately 34.7% and 66.1% decrease in concentration of the free sulfhydryl groups, respectively, indicating that the encapsulated BSA likely reacted with the P407-diacrylate polymer (Figure S3).

After day 49, both cross-linked hydrogels exhibited an accelerated release, which can be attributed to degradation of the hydrogel. Interesting to note is that the cumulative release of BSA from the P407-10K gels began to surpass P407-20K gels, reaching 43.5 ± 0.1% at day 56 and 94.7 ± 1.0% at day 63. In contrast, the slow cumulative release of BSA from the P407-20K gels continued for an additional 7 days, achieving 36.2 ± 0.1% at day 56, 63.1 ± 0.2% at day 63, and 91.3 ± 2.2% at day 70. The faster release rate observed in P407-10K gels compared to P407-20K gels at later time points can be attributed to the percentage difference in the BSA chemically tethered to the P407-diacrylate polymer (Figure S3) and the difference in PEG cross-linker weight concentration between the two hydrogels, which will be further elaborated in Section 3. Overall, cumulative release profile results confirm that secondary chemical cross-linking in P407-10K gels and P407-20K gels enables prolonged release of BSA compared to P407 gels.

### 2.4 Stability and Biophysical Characterization of BSA Released from P407-10K and P407-20K Gels

The structural integrity of BSA released from P407-10K gels and P407-20K gels was then assessed using reduced capillary electrophoresis-sodium dodecyl sulfate (CE-SDS, Figure 4a and Figure S4) along with a control BSA sample where 40 mg/mL of BSA was incubated in 1× PBS incubated at 37 °C. As of note, to remove heterogeneous hydrogel fragments from the release media of the P407-10K gels and P407-20K gels (without BSA encapsulated), as indicated by size exclusion chromatography (SEC) (Figure S5), BSA was purified using the acetone precipitation method, since these hydrogel fragments could interfere with the biophysical characterization process. On day 1, the monomer percentage of BSA released from both P407-10K gels and P407-20K gels was similar to that of the control, indicating that these BSAs were likely to be those that are loosely bound to the hydrogel surface through nonspecific absorption. From days 7 to 28, the monomer fractions of BSA released from the gels were higher than those of BSA control, possibly suggesting that the hydrogels effectively delayed aggregation consistent with literature findings that hydrogels can stabilize biologics by encapsulating proteins in a three-dimensional network, limiting their mobility and reducing aggregation.^55–57^ During this period, approximately only 8.1% and 11.2% of BSA were released from P407-10K gels and P407-20K gels, respectively (Figure 3b). These BSA fractions were likely to be released via diffusion and do not bind to the P407-diacrylate polymer. Starting from day 49, the BSA monomer fraction decreased with reduced monomer fractions in both P407-10K gels and P407-20K gels compared to the BSA control. Heat-induced aggregation was ruled out as no significant aggregation of BSA control was observed (Figure 4a and Figure S4c) and BSA having a melting temperature of 63 °C.^58^ Hence, the decrease in monomer fraction at later release time points can be attributed to the BSA fractions that could have reacted with the P407-diacrylate polymer, as BSA contains a free sulfhydryl group. SEC analysis revealed high molecular weight fractions in the released BSA from hydrogels (Figure S6a,b) that were not observed in the BSA control (Figure S6c), indicating that these fractions are tethered to the P407-diacrylate polymer and are likely released through hydrogel degradation rather than by diffusion.

**Figure 4:**
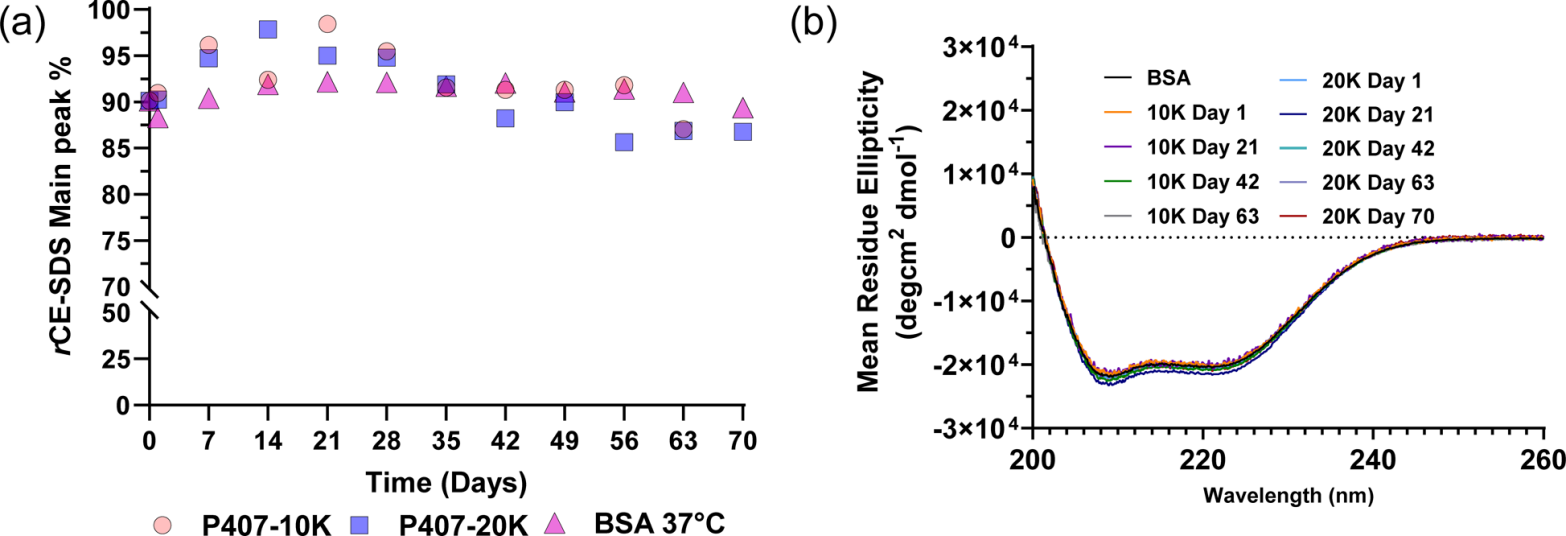
(a) Main peak percentage of BSA in reduced CE-SDS released from P407-10K gels, P407-20K gels, and BSA solution at 1× PBS, pH 7.4 at 37 °C during a 70 day release/incubation period at different time points (*n* = 1). (b) CD spectroscopy of BSA released from P407-10K gels and P407-20K gels in 1× PBS, pH 7.4 at 37 °C at different time points (*n* = 3).

Circular dichroism (CD) spectroscopy analyses were also conducted at 3 week intervals throughout the release duration to assess any changes in the secondary structure of the released BSA. BSA released from P407-10K gels and P407-20K gels both exhibited negative bands around 208 nm and 222 nm, which are indicative of an α-helical structure with no significant changes during the 70 days of the release study (Figure 4b). From the overall biophysical and stability characterization results, despite the majority of the BSA encapsulated within the cross-linked P407 hydrogels retained its conformation throughout the entire release duration from the hydrogels, caution is warranted encapsulating free sulfhydryl group containing biologic due to potential interactions with the P407-diacrylate polymer that might hinder the release of biologics and alter their biophysical properties.

### 2.5 Release Kinetics of Adalimumab from P407-10K Gels and P407-20K Gels

To assess the versatility of the cross-linked P407 hydrogels for various therapeutic modalities, the release profiles of a therapeutic anti-TNF alpha monoclonal antibody (mAb) adalimumab (recombinant IgG1, ∼148 kDa) were also studied after encapsulated within the hydrogels (20 mg/mL adalimumab). Attempts to encapsulate adalimumab at higher concentrations were avoided because the commercial liquid formulation was 100 mg/mL and concentrating it further (by spin-filter concentration or lyophilization) could risk compromising the antibody’s structural integrity during hydrogel encapsulation. In the P407 hydrogels, a zero-order release profile was exhibited where complete release of adalimumab was achieved within 24 h (Figure 5a). However, in the chemically cross-linked P407 hydrogels, adalimumab was released over 70 days. P407-10K gels exhibited a triphasic release profile, whereas P407-20K gels exhibited a biphasic release profile (Figure 5a). For P407-10K gels, a cumulative release of 13.4 ± 2.5% was observed during the initial burst release phase over the first 4 days. In the second phase, P407-10K gels exhibited a slow-release plateau up to day 21, with a cumulative release of 15.0 ± 2.5%. The third phase of P407-10K gels exhibited an accelerated but still sustained release profile until the completion by day 63. For P407-20K gels, an initial burst was observed, with a cumulative release of 19.3 ± 2.8% for the first 4 days. The second phase exhibited a constant sustained release that continued until completion by day 70.

**Figure 5:**
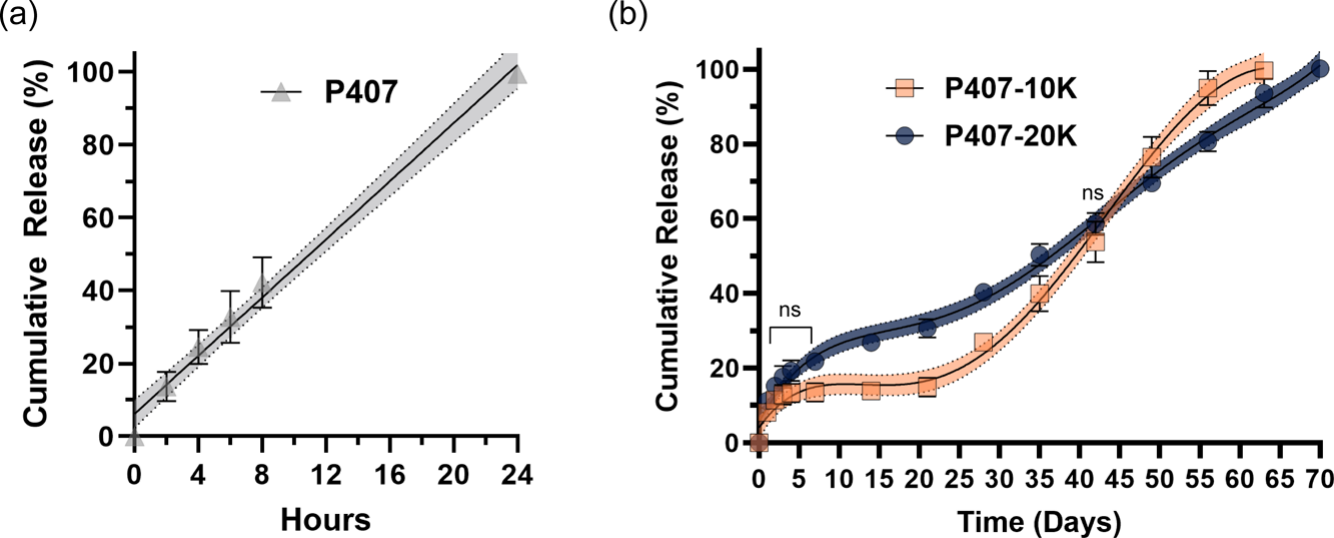
Cumulative release of adalimumab from (a) P407 and (b) P407-10K and P407-20K hydrogel formulations in 1× PBS, pH 7.4 at 37 °C (average ± S.D, *n* = 3) with 95% confidence band. Repeated measures analysis revealed that the cumulative release percentage of adalimumab between P407-10K gels and P407-20K gels is statistically significantly different from one another, except for days 1 to 7 and day 42 (*p* < 0.05).

Between days 7 and 42, the cumulative adalimumab release profile of P407-20K gels was significantly faster compared to that of the P407-10K gels, as verified by repeated measure analysis (Table S4). Such difference can be explained by the different MW of the 8-arm-PEG-SH cross-linker used in the hydrogel composition. An increased molecular weight of the cross-linker results in decreased cross-linking density, allowing for a higher gel swelling ratio (Figure 2a,b), which facilitates the diffusion of monoclonal antibodies out of the hydrogel network. After day 21, the antibody release profile of the P407-10K gel accelerated, with a crossover point at day 42, surpassing the cumulative release percentage of the P407-20K gel by day 49 (P407-10K gel cumulative release: 76.5 ± 5.4% and P407-20K gel cumulative release: 69.5 ± 1.3%). Comparable in size to adalimumab, the human serum IgG antibody (MW ∼150 kDa) was also encapsulated in both P407-10K gels and P407-20K gels. Both the P407-10K gels and P407-20K gels exhibited a release profile similar to that of the adalimumab release profile (Figure S7 and Table S5). Comparable mAb release data was reported by Gregoritza et al. where cross-linked poloxamine (Tetronic 1107) hydrogels exhibited a triphasic release profile of bevacizumab with variable release duration depending on the cross-linking density.^38^ Similar to BSA release kinetics, P407-10K gels exhibited an accelerated release rate of adalimumab at later time points compared to P407-20K gels. This is noteworthy despite the swelling ratio of P407-20K gels being significantly higher than that of P407-10K gels during the same period (Figure 2b and Table S1). Additionally, differences in the reduction of release media volume withdrawn from the cross-linked hydrogels were observed over time, confirming the difference in the hydrogel swelling ratio between P407-10K gels and P407-20K gels. Specifically, for the P407-20K gels, the volume decreased from 3 mL to 2.5 mL between day 1 and day 70. In contrast, the volume for the P407-10K gels decreased from 3 mL to 2.7 mL from day 1 to day 63. The accelerated release rate of adalimumab in P407-10K gels, despite the lower swelling ratio compared to P407-20K gels, can also be attributed to the lower concentration of the PEG cross-linker in the P407-10K gels compared to P407-20K gels, a point that will be further elaborated in the discussion section

### 2.6 Stability and Biophysical Characterization of Adalimumab Released from Cross-Linked P407 Hydrogels

Biophysical characterization of adalimumab released from hydrogels was then assessed using size exclusion chromatography (SEC) and capillary electrophoresis of sodium dodecyl sulfate (CE-SDS) to assess potential aggregation or fragmentation. For these experiments, control samples included adalimumab stock stored at −80 °C (referred to as “adalimumab −80 °C” in Figures S8, S10, and S11) and 20 mg/mL adalimumab solution that was incubated at 37 °C for 70 days (referred to as “adalimumab 37 °C”). As of note, to remove heterogeneous hydrogel fragments from the release media of the P407-10K gels and P407-20K gels (Figure S5), a protein A affinity spin column was employed to purify adalimumab. Adalimumab released from P407-10K gels and P407-20K gels maintained 89–98% monomer content, similar to the 37 °C control, indicating that the released adalimumab remained primarily in a monomeric form (Figure 6a, Figure S8a,b). Previous existing studies have noted that the thermal stress can reduce monomer percentages due to high molecular weight (HMW) aggregates or low molecular weight (LMW) species forming under heat-induced unfolding conditions, which expose hydrophobic regions and promote aggregation.^59–62^ Additionally, although PEG can induce protein precipitation,^42,63^ our results confirm that the monomer fraction during release remained comparable to 37 °C control, indicating that aggregation due to hydrogel encapsulation or PEG induced precipitation was minimal (Figure 6a and Figure S8c).

**Figure 6:**
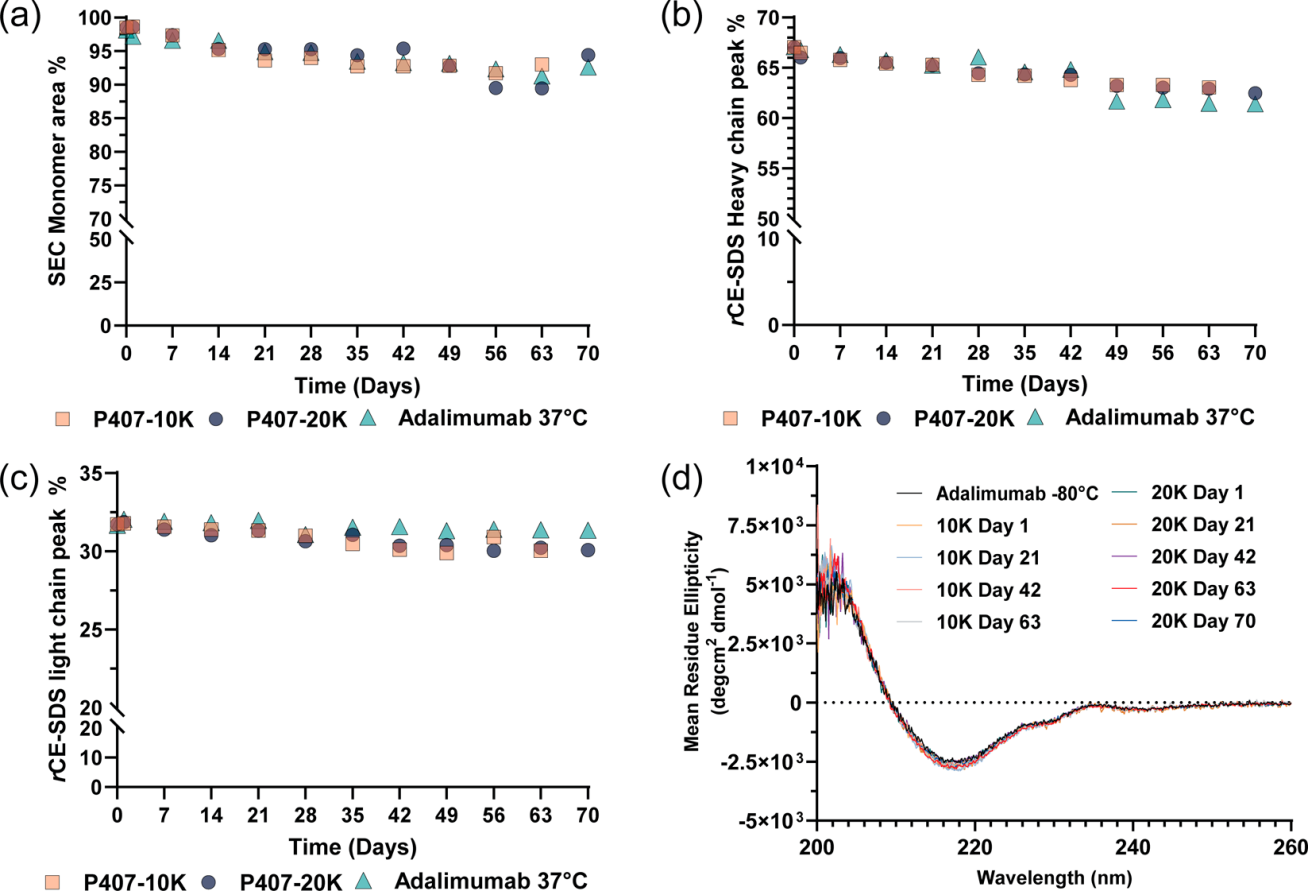
Biophysical characterization of adalimumab released from P407-10K gels and P407-20K gels in 1× PBS, pH 7.4, at 37 °C. (a) Main peak percentage of adalimumab in SEC, (b) heavy chain peak percentage of adalimumab in reduced CE-SDS (*n* = 1), and (c) light chain peak percentage of adalimumab quantified from P407-10K gels, P407-20K gels, and adalimumab solution at 37 °C during a 70 day release/incubation period at different time points (*n* = 1). (d) CD spectroscopy of adalimumab released from P407-10K gels and P407-20K gels at different time points (*n* = 3).

As a complementary analysis, nonreduced CE-SDS revealed a less than 10% decrease in intact adalimumab over the release period compared to the −80 °C control (Figures S9 and S10a,b). Consistent with the SEC results, there was an increase in HMWs as well as LMWs, particularly heavy–heavy (HH) and heavy–heavy-light (HHL) species, likely due to disulfide bond scrambling under heat stress conditions.^64^ 37 °C control also exhibited similar increase in in HH and HHL species over incubation time, indicating that hydrogel encapsulation did not induce any significant aggregation or fragmentation, consistent with SEC findings (Figure S10c). To further analyze the intact heavy chain and light chain fraction in the adalimumab, reduced-CE-SDS analysis showed less than 5% decrease in the heavy chain fraction and less than 1% decrease in the light chain fraction compared to the −80 °C control, suggesting the structural integrity of adalimumab was largely maintained through the release duration (Figure 6b,c, Figure S11a,b). Small peaks observed at relative migration time (RMT) at 1.363 at 1.466 (Figure S11a,b) likely represent fragmentation products due to peptide bond hydrolysis,^65–67^ while the peak at RMT 1.783 would likely correspond to nonreducible aggregates possibly formed by isopeptide bond formed between amino group of lysine residues and the carboxyl group of aspartic acid or glutamic acid residues or oxidation-induced dimerization of tyrosine residues.^68,69^ Notably, reduced CE-SDS results showed an increase in nonreducible aggregates in adalimumab encapsulated in P407-10K and P407-20K hydrogels compared to the “adalimumab 37 °C” control, with the most significant differences observed at later time points (Figure S11). Specifically, peak percentages of adalimumab at 37 °C were both 2.1% on day 63 and day 70, while those in P407-10K gels and P407-20K gels were both 4.0%. We hypothesize that the slightly higher levels of nonreducible aggregates in the hydrogels result from prolonged exposure to the hydrogel microenvironment, where changes in local pH and ionic strength may enhance amino acid reactivity, promoting isopeptide bond formation, and reduced oxygen levels could facilitate oxidation-induced dimerization.^68,69^ Despite the increase in aggregates, the “adalimumab 37 °C” control maintained similar heavy and light chain percentages over the 70 day incubation period (Figure 6b,c and Figure S11c), indicating that the cross-linked P407 hydrogels did not significantly induce degradation or fragmentation of adalimumab.

Finally, circular dichroism (CD) spectroscopy analysis was also conducted at three-week intervals throughout the release duration to assess changes in the secondary structure of released adalimumab. Both adalimumab samples released from P407-10K gels and P407-20K gels exhibited a negative peak near 217 nm and maximum peak near 202 nm, which is indicative of a secondary structure with β sheet as reported in the literature (Figure 6d).^70,71^ Most importantly, the CD spectra of the released adalimumab from both hydrogels were almost identical to the adalimumab −80 °C control throughout the entire release duration, indicating that the conformational integrity of adalimumab was well preserved during hydrogel encapsulation and release.

### 2.7 Functional Characterization of Released Adalimumab from P407-10K Gels and P407-20K Gels

To examine the biological potency of adalimumab released from P407-10K gels and P407-20K gels, a competitive TNFα: human TNF receptor 1 enzyme-linked immunosorbent assay (ELISA) was conducted. The half maximal inhibitory concentration (IC50) of adalimumab released from P407-10K gels at day 63 and P407-20K gels at day 70 was determined. Control groups included adalimumab stock stored at −80 °C (referred to as “adalimumab −80 °C” in Figure 7a,b) and 20 mg/mL adalimumab solution incubated at 37 °C for 70 days (referred to as “adalimumab 37 °C day 70” in Figure 7a,b). The IC50 values of the adalimumab released from P407-10K gels and P407-20K gels were 254.1 ± 50.7 ng/mL and 231.0 ± 32.1 ng/mL, respectively (Figure 7a). Compared to the IC50 values of the “adalimumab −80 °C” and “adalimumab 37 °C day 70” control groups, which were 224.2 ± 46.0 ng/mL and 249.0 ± 49.3 ng/mL, respectively, the results confirm that the potency of the monoclonal antibody was not affected by the hydrogel cross-linking and its extended encapsulation within the P407-10K gels and P407-20K gels. Despite an ∼10% decrease in the monomer fraction of adalimumab released from cross-linked hydrogels at days 63 and 70, compared to “adalimumab −80 °C” control (as verified by SEC and nonreduced CE-SDS), the functionality of the released adalimumab did not show any statistically significant difference (*p* > 0.05) when comparing the means of IC50 between all four groups when analyzed by one-way analysis of variance (ANOVA) followed by Tukey’s multiple comparison test (Figure 7b, Table S6). Similar results were noted by Heljo et al., which observed no significant IC50 changes with a 2.2% decrease in monomer fraction due to heat and stirring conditions.^72^ A significant IC50 reduction (50.7%) occurred only under strong oxidative stress condition, which caused a 64.4% decrease in the monomer fraction due to post-translational modifications.^72^

**Figure 7:**
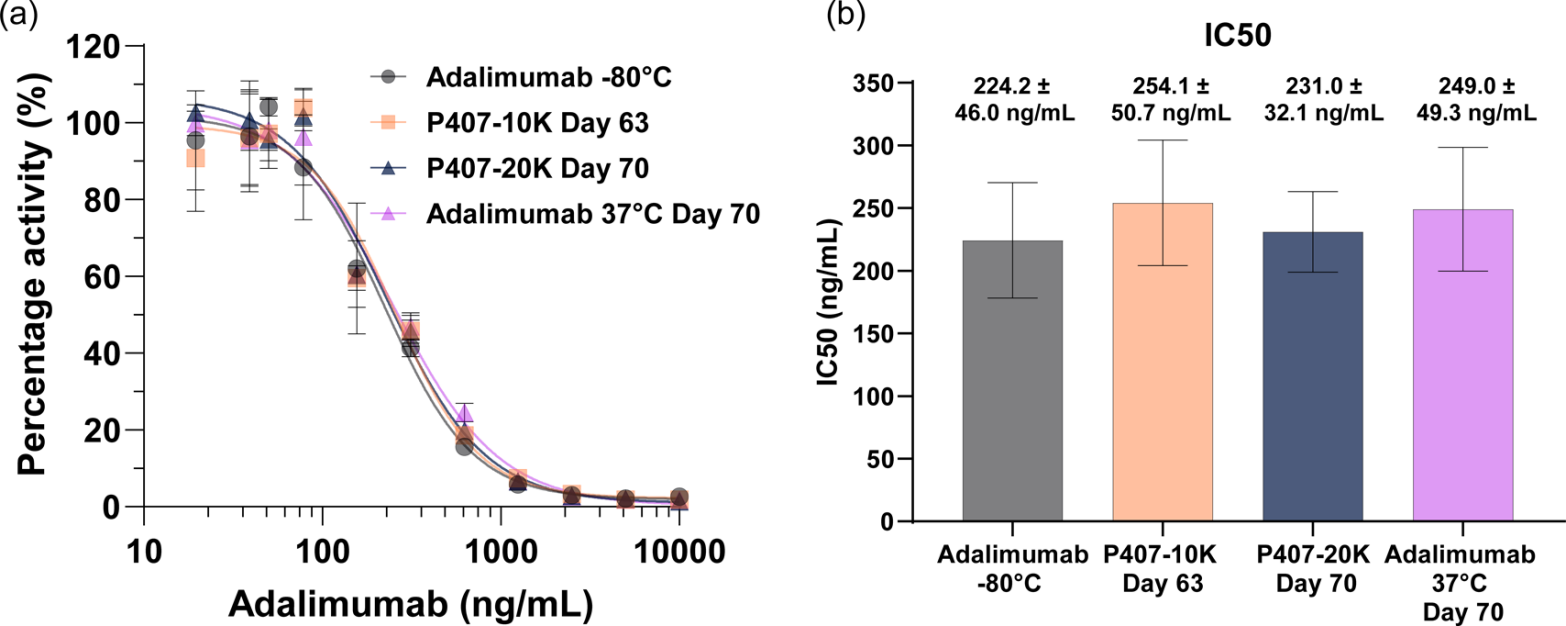
(a) Inhibition of profiles of adalimumab by TNF-α: human TNFR1 blockade competitive ELISA assay. The absorbance at 412 nm was normalized to negative control (no adalimumab added to wells) as a percentage of activity. (b) Calculated IC50 values are provided for each adalimumab groups (*n* = 3, average ± SD). When comparing the mean IC50 values, there was no statistically significant difference between all groups (*p* > 0.05) when analyzed by ordinary one-way analysis of variance (ANOVA) with Tukey’s multiple comparison test.

## 3 Discussion

In this study, P407 hydrogels were formulated using 10 and 20 kDa 8-arm-PEG-SH linkers via Michael-type addition to improve mechanical strength and extend the release duration of encapsulated biologics. In an in vitro setting at 1 × PBS (pH 7.4) at 37 °C, both of the chemically cross-linked P407-10K and P407-20K were able to achieve complete release of encapsulated biologics in a 70 day time frame. Two factors that can contribute to complete release of biologics would be increased hydrogel swelling over time, as described in Figure 2b and biodegradation of the hydrogel driven primarily by hydrolysis of ester linkages. As shown in Scheme 1, the P407-diacrylate used to formulate P407-10K and P407-20K gels contains acrylate-ester linkages (CH_2_=CH–CO–O-P407–O–CO–CH=CH_2_) that are hydrolytically labile. Incubation of these chemically cross-linked hydrogels in PBS (pH 7.4) at 37 °C for 70 days is expected to undergo hydrolysis of those ester bonds and result in gradual disintegration of the hydrogel. A similar report has been reported in hydrogel chemically cross-linked via Michael addition: PEG400-diacrylate cross-linked a with 3-arm-thiol polymer gradually degraded over 10 weeks, with degradation attributed primarily to hydrolysis of the ester linkage incorporated in the PEG400-diacrylate. Although the polymers differ in molecular weight, the dominant degradation mechanism for the chemically cross-linked P407 hydrogels is likewise expected to be hydrolysis of the ester bond.

Similarly, biodegradation of chemically cross-linked P407 hydrogels in vivo is expected to proceed primarily by hydrolysis of ester linkages and oxidation of thioether bonds. Because the acrylate groups are attached to the polymer by ester bonds, network cleavage will occur via ester hydrolysis and can be accelerated by esterases present in serum and tissues. Thioether bonds formed by thiol-acrylate Michael addition are relatively resistant to hydrolysis but are susceptible to oxidation in vivo. Reactive oxygen species produced by activated inflammatory cells (e.g., neutrophils and macrophages) can oxidize thioethers to sulfoxides and sulfones, which can alter network integrity and accelerate degradation locally, particularly in inflamed tissues.

Our expected target product profile of such a hydrogel system includes the use of two syringes (Figure S12), where biologics are formulated in P407-diacrylate polymer containing solution in one syringe and 8-arm-PEG-SH in another. This setup allows for cold chain logistics during transport, with components mixed immediately at ambient temperature before injection, which can enable formation of an in situ hydrogel upon injection at physiological temperature.

In contrast to P407 gels, which achieve gelation within 5 min (Figure 1c), P407-10K and P407-20K gels exhibited slower gelation, taking about 30 min after the temperature is raised from 4 to 37 °C (Figure S2). We hypothesize that several factors contribute to these slow gelation kinetics. First, chemical cross-linking with 8-arm-PEG-SH would restrict the mobility of the P407-diacrylate polymer chains, unlike P407 gels, in which the P407 polymer chains can move freely to accelerate micelle formation leading to faster gelation. Additionally, the cross-linked hydrogel network would increase viscosity and rigidity, impeding the flow and rearrangement of both the P407-diacrylate polymer chains and the 8-arm-PEG-SH linkers. Consequently, the complete chemical cross-linking process is delayed, as indicated by the storage modulus (*G*′) not yet reaching a plateau (Figure S2). Regarding injectability, the 8-arm PEG cross-linker solutions are highly soluble in water and have a suitable viscosity for injection. A previous study demonstrated that a 25% P407 solution behaves like a liquid at 18 °C, and its viscosity (cP) does not significantly increase at ambient temperatures below 20 °C, suggesting that it remains injectable under typical conditions.^19^ Based on the gelation kinetics data (Figure S2) and previous reports, the proposed target product profile (Figure S12) would be considered feasible

Through strategic integration of the thermal gelling properties of P407 and spontaneous, physiologically relevant Michael-type addition, these cross-linked P407 hydrogels can form an in situ gel upon injection while gradually reinforcing the network through chemical cross-linking and extend the release duration of encapsulated biologics. Remarkably, the release durations of chemically cross-linked P407-10K gels and P407-20K gels that encapsulated BSA were extended up to 70 days, compared to P407 gels, which released BSA over just 24 h. When encapsulating BSA, a slower release kinetics profile was observed, where cumulative release was less than 22% for both hydrogel groups tested up to day 42. The free sulfhydryl group in BSA contributed to its retention in the hydrogel by reacting with a P407-diacrylate polymer. This interaction was confirmed by a reduction in free sulfhydryl groups in BSA encapsulated within cross-linked P407 hydrogels, as determined by Ellman’s assay (Figure S3).^45^ The possibility of interaction between BSA and 8-arm-PEG-SH was ruled out, as the thiol-acrylate reaction is significantly faster due to the inherent nucleophilicity of the thiol group and the electrophilic nature of the acrylate double bond.^39^ Consequently, the thiol-acrylate reaction is a much more rapid chemical process compared to disulfide exchange reactions.^73,74^ Therefore, caution should be exercised when incorporating biologics containing free sulfhydryl groups, unless the goal is to covalently conjugate the biologic to the hydrogel while ensuring that the encapsulated biologic retains its functionality. In our study, BSA was used as a model molecule; however, in the development and manufacturing process of therapeutic proteins, reactive cysteine residues, especially those exposed on the protein surface, are capped through various engineering methods.^75,76^ Although covalent interactions with the payload may not always be desirable, they can be strategically leveraged to enhance the functionality and versatility of hydrogel systems when appropriately selected molecules are employed for specific indications. Previous studies by Hubbell et al., Heilshorn et al., and other groups have demonstrated that the covalent conjugation of matrix metalloproteinase (MMP),^77,78^ vascular endothelial growth factor (VEGF),^79^ epidermal growth factor (EGF),^80^ transforming growth factor 1 β1 (TGF-β1),^81^ bovine IgG,^82^ bevacizumab,^82^ and ranibizumab^82^ can be advantageous for drug delivery and tissue engineering applications.

The cumulative release profiles of adalimumab encapsulated in P407-10K gels and P407-20K gels reveal distinct behaviors: P407-10K gels exhibits a triphasic release profile, while P407-20K gels exhibits a biphasic release profile. The initial burst of adalimumab release during the first week, from day 1 to day 7, can be attributed to the drug being loosely bound to the gel’s surface through nonspecific absorption. Consistent with the results of the repeated measures analysis, there was no statistically significant difference in the cumulative release of adalimumab between the P407-10K and P407-20K gels during this period (Table S5).

In the subsequent phase, both P407-10K and P407-20K gels exhibited slower release behavior. This slower release is likely governed by diffusion, influenced by the molecular weight (MW) of the cross-linker used. Notably, the cumulative release of adalimumab was significantly higher in the P407-20K gels compared to the P407-10K gels, up until day 35, which aligns with the higher swelling ratio of P407-20K gels compared to P407-10K gels (Figure 2b). Although no significant difference was observed in the swelling ratio between P407-10K gels and P407-20K gels from day 14 to day 35 (Table S1), it is important to consider the hydrodynamic diameter of adalimumab, which is reported to be between 11.2 and 11.8 nm.^83^ This diameter is larger than the initial mesh sizes of the cross-linked hydrogels, which are approximately 6.2–6.8 nm for P407-10K gels and 6.4–7.3 nm for P407-20K gels at 37 °C (Table S2). The larger hydrodynamic diameter of adalimumab may restrict its rapid diffusion from the P407-10K gels compared to the P407-20K gels. This suggests that the mesh size of the hydrogel at early time points may limit the diffusion of adalimumab, which could help explain the significant difference in the cumulative release of adalimumab between P407-10K gels and P407-20K gels during days 7 and day 42. As the time went further, P407-10K gels started to swell more, which explains the reason for no statistical significance between the swelling ratio of P407-10K and P407-20K from day 14 to day 35 (Table S1).

Notably in our study, we observed a crossover (between days 42 and 49) in the cumulative lease kinetics of various biologics such as BSA, human serum IgG, and adalimumab. The cumulative release at the time point following the crossover was statistically significant, as confirmed by repeated measures analysis (Tables S3–S5). Despite the statistically higher swelling ratio of P407-20K compared to P407-10K at later time points after day 42 (as determined by repeated measures analysis, Table S1), the cumulative release percentage of P407-10K exceeded that of P407-20K after day 49. This finding contrasts with reports suggesting that the swelling ratio and release rate of biologics are inversely correlated.^46,47^ We hypothesize that the faster release rate observed in P407-10K gels compared to P407-20K gels at later time points can be attributed to the differences in PEG cross-linker weight concentration. In our study, P407 hydrogels were chemically cross-linked using P407-diacrylate polymer and 8-arm-PEG-SH (10 or 20 kDa) at a one-to-one stoichiometric ratio between thiol and acrylate groups. This ratio was chosen instead of normalizing to the same weight percentage of 8-arm-PEG-SH cross-linkers in the hydrogel system to prevent any excess free thiol groups, which could potentially interact with and destabilize the encapsulated biologics over time within the hydrogel matrix. Specifically, 25 mg of 8-arm-PEG10K-SH was added to 0.5 mL of a 25% P407-diacrylate solution, while 50 mg of 8-arm-PEG20K-SH was used in the same volume to maintain a 1:1 thio:acrylate molar ratio. As a result, the PEG cross-linker concentration in the P407-10K gel (5.0 wt %) is half that of the P407-20K gel (10.0 wt %). Initially, the higher cross-linking density in P407-10K gel leads to slower release of encapsulated biologics due to the shorter PEG arm length. However, at later time points, the hydrogels absorb aqueous solution and swell (Figure 2b), increasing their total volume. We hypothesize that the lower PEG concentration in P407-10K gel enhances the diffusivity of the encapsulated biologics, facilitating a more rapid release consistent with multiple literature indicating that decreased PEG weight concentration correlates with increased release rates by reducing network density and steric hindrance, which facilitates higher molecular mobility.^43,47,53,84,85^

Prolonged release of encapsulated biologics from the cross-linked P407 hydrogels can ensure a steady release of a therapeutic agent over time, reducing the need for frequent dosing, improving patient compliance. Different release profiles were achieved by varying the molecular weight of the linker, which can be further modulated by varying cross-linker’s molecular weight, branching arm number, or the thiol-acrylate stoichiometry. Control over the timing and amount of drug released can be tailored to meet specific therapeutic needs. The initial burst release phase facilitates rapid drug delivery, achieving immediate therapeutic effects. The second phase provides a steady and controlled release of the drug over an extended period, which can be beneficial for maintaining therapeutic drug levels in the bloodstream, reducing the frequency of dosing, and improving patient compliance. In the third phase, as the hydrogel matrix begins to erode, the drug release rate increases again. This can be advantageous in treatments requiring a higher concentration of the drug during later phases in therapies such as human growth hormone (HGH) therapy for children with growth hormone deficiency^86,87^ and interferon beta 1a therapy for patients with relapsing multiple sclerosis.^88,89^ Another potential application of the triphasic release profile is vaccine delivery, where the intermittent release could provide pulsed antigen dosing from a single administration, mimicking traditional multidose regimens and enhancing immune response.^90–92^ For biphasic release profiles, the sustained release following the initial burst can be particularly important for chronic conditions where long-term treatment is necessary and helping to maintain levels within the therapeutic range while avoiding dosage that could lead to toxicity, such as potent cytokines and small molecule inhibitors for immunotherapy and oncology applications.^9,93,94^

Although many studies have sought to improve the mechanical strength and extend the release of P407-based hydrogels, this study also aimed to explore the structural integrity and conformational properties of encapsulated proteins during the incubation and release processes. Biophysical analyses—including size exclusion chromatography (SEC), capillary electrophoresis-sodium dodecyl sulfate (CE-SDS), and circular dichroism (CD)—revealed that the cross-linked P407 hydrogels did not adversely affect the stability of encapsulated adalimumab throughout the encapsulation process and during the 70 day release period at physiological temperature (37 °C). This finding was further supported by the maintained biological function, as assessed by a competitive ELISA assay. Cross-linking the P407 hydrogels not only helps prevent burst release but also extends the duration of biologic release with tunable kinetics. Additionally, this study provides valuable insights into the biophysical stability profile of the proteins, which may be relevant for the clinical translation of long-acting hydrogel formulations of biologic therapeutics.

While the proposed cross-linked P407 hydrogel system via Michael-type addition in vitro is promising, it could be further enhanced with orthogonal cross-linking chemistries for better selectivity and robustness of gelation in the biological context. The in vivo administration process would involve simultaneously mixing P407-diacrylate polymer solution with the biological component and the cross-linker fraction right before application. However, in situ gelation can be challenging due to temperature variations and different pH environments encountered in the body as well as potential undesired interactions with the encapsulated biologic cargos. Especially, low pH, which is more commonly associated with various disease conditions such as the tumor microenvironment or inflamed tissues, can significantly alter gelation kinetics. The thiol-acrylate Michael addition reaction is particularly sensitive to pH, with significantly reduced reaction rates at lower pH values.^95^ Eliyahu et al. demonstrated this effect in hydrogels formed by thiol-acrylate reactions, where some acrylate groups remained unreacted even after 72 h at pH 4 and pH 5.6.^96^ Hence, the proposed cross-linked P407 hydrogel might face difficulties in forming a stable gel structure in low pH in vivo conditions, potentially leading to premature dissolution and release of the cargo. Hence, an alternative cross-linking with suitable reaction kinetics could be more suitable for low pH conditions. For instance, copper-free strain promoted alkyne-azide cycloaddition (SPAAC)^97^ and tetrazine-trans-cyclooctene ligation (TCO ligation),^98^ utilizing strained alkynes and azides or tetrazines and TCO, respectively, generally exhibit stable reaction rates across a broad pH range, making them less sensitive to pH variations in specific in vivo conditions.^99^ Moreover, this cross-linking chemistry could eliminate potential interaction between the free sulfhydryl group containing biologics and the acrylate group. To avoid potential interaction between the free thiol groups and the acrylate group in P407, other alternative cross-linking chemistries can also be employed. These include the Diels–Alder reaction,^100^ which utilizes diene bonds in conjunction with an alkene, oxime ligation^101^ involving aminoxy group and a carbonyl groups (aldehyde or ketone), Schiff-base cross-linking reaction^102^ that engages primary amine and carbonyl group, host–guest interactions^103^ utilizing adamantane and cyclodextrin, and the formation of boronic ester bonds using boronic acids and 1,2-diol or 1,3-diol structures.^55^ More investigations of the incorporating alternative chemistries to enable P407 cross-linking will be a subject of future report.

Overall, our work uniquely leverages the in situ gelation of thermoresponsive P407 as well as Michael-type addition as a secondary cross-linking mechanism to address P407’s short in situ residence time and to prolong release of encapsulated biologics. While prior studies by Hubbell et al. primarily focused on using this cross-linking methodology for cell encapsulation,^34,35^ we extended this strategy to chemically cross-linked P407 hydrogels to specifically improve gel stability and achieve sustained release of biologics over extended periods. To our knowledge, there are few reports of P407 formulations that maintain controlled biologic release beyond 1 week; here, we demonstrate sustained release well past that time frame, highlighting the novelty of this approach. The PEG cross-linker’s molecular weight, branching arm-number, or weight concentration can be modulated to fine-tune the release profiles. Cross-linking the P407 gels via photopolymerization can provide rapid gelation and high spatial control,^28–30^ but it requires light exposure and often the use of photo initiators, which can cause potential protein damage or denaturation. Chemical cross-linking via Michael-type addition at physiological conditions would reduce the risk of phototoxicity while preserving the biologic’s integrity during gelation. One potential limitation of the Michael-type addition would be undesired covalent interactions with the payloads that contain endogenous thiols, which necessitate caution in payload selection and understanding of protein structures and reactive functional groups.

## 4 Conclusions

Thermoresponsive in situ hydrogels have emerged as a promising platform for the minimally invasive delivery of biologics. P407 has garnered considerable attention; however, its weak mechanical strength and rapid dissolution have limited its use for sustained release of therapeutics. This work established a facile method to enhance hydrogel stability by utilizing diacrylate modified P407 and an 8-arm-PEG-thiol cross-linker through Michael-type addition. Specifically, this study has demonstrated that chemically cross-linked P407 hydrogels can achieve an extended release of biologics for up to 70 days, compared to non-PEG cross-linked hydrogels, with tunable release kinetics depending on the molecular weight of the cross-linker used. Biophysical characterization of the encapsulated cargoes using SEC, CE-SDS, and CD was conducted throughout the entire release period. Most importantly, the functionality of the encapsulated biologic remained unaffected by the hydrogel cross-linking preparation and the extended release at physiological temperature (37 °C). The results of our study provide valuable insights for developing long-acting injectable formulations that can be precisely tailored to meet the demands of various therapeutic applications by adjusting the cross-linker’s molecular weight, branching arm-number or weight concentration to modulate the release kinetics profile.

## 5 Experimental Section

### 5.1 Materials

Poly­(ethylene oxide)-*b*-poly­(propylene oxide)-*b*-poly­(ethylene oxide) diacrylate end-cap (P407-diacrylate, MW ∼12,500) was purchased from Polyscitech (AI146, Akina Inc.). Eight-arm, thiol-functionalized PEG (8-arm-PEG10K-SH, Mn: 10,000 and 8-arm-PEG10K-SH, Mn: 20,000) were purchased from JenKem Technology USA Inc. (Allen, TX). Bovine serum albumin (BSA) was obtained from Sigma-Aldrich (St. Louis, MO). Immunoglobulin G from human-plasma (IgG, MyBioSource) was purchased from Fisher Scientific (Hampton, NH). Adalimumab was purchased from Eurofins (Lancaster, PA). All other reagents and materials were purchased from Fisher Scientific, unless otherwise mentioned.

### 5.2 Formulation of Chemically Cross-Linked P407 Hydrogels

Chemically cross-linked P407 hydrogels were formulated by a Michael-type addition between multiarm PEG-SH and P407-diacrylate polymer. P407-diacrylate polymer (2500 mg) was weighed in 20 mL glass vial, and 10 mL of phosphate buffered saline (1× PBS, pH 7.4) was added to make 25 wt % (weight percent) solution. The mixture was stirred overnight at 4 °C to ensure complete solubilization of the of P407-diacrylate polymer. P407-diacrylate polymer solution (0.5 mL) was then aliquoted to a 4 mL glass vial at 4 °C. For P407-10K hydrogel, 25 mg of 8-arm-PEG10K-SH was added to the dissolved polymer solution. For the P407-20K hydrogel, 50 mg of 8-arm-PEG20K-SH was added to the dissolved polymer solution. For both cases, the molar ratio between thiol and acrylates was 1:1. After confirming the PEG cross-linkers were fully dissolved within the P407-diacrylate polymer solution, the vial was transferred to a 37 °C incubator and was left overnight to achieve maximum cross-linking. For the encapsulation of BSA, 20 mg of lyophilized BSA powder (Sigma-Aldrich, St. Louis, MO) was directly added to the polymer-cross-linker mixture at 4 °C after confirming the PEG cross-linkers were fully dissolved within the polymer solution to achieve a final concentration of 40 mg/mL. For P407 hydrogel, 0.5 mL of 25 wt % P407 polymer solution was mixed with 20 mg of lyophilized BSA powder at 4 °C. After confirming the lyophilized BSA powder is fully solubilized in the polymer-cross-linker mixture, the vials were then transferred to a 37 °C incubator and were left overnight to ensure full chemical coss-lirnking before initiating in vitro release experiment. For encapsulation of human plasma IgG, 200 mg of lyophilized IgG powder was directly added to the polymer-cross-linker mixture at 4 °C to achieve a final concentration of 100 mg/mL. For encapsulation of adalimumab, 31.25 wt % P407-diacrylate polymer solution was prepared. When formulating the cross-linked hydrogel, 0.4 mL of 31.25 wt % P407-diacrylate polymer solution was added with 25 mg of 8-arm-PEG10K-SH or 50 mg of 8-arm-PEG20K-SH was added to form P407-10K and P407-20K hydrogels, respectively. After the PEG cross-linkers were fully dissolved, 0.1 mL of 100.0 mg/mL of adalimumab solution (total of 10 mg) was added to achieve a final protein concentration of 20 mg/mL and final wt % of P407-diacrylate polymer solution to be 25 wt %. For the P407 hydrogel, 0.4 mL of 31.25 wt % P407-diacrylate polymer solution was mixed with 0.1 mL of 100.0 mg/mL of adalimumab solution without any cross-linker. After confirming the cross-linker was fully solubilized in the polymer-cross-linker mixture, the vials were then transferred to a 37 °C incubator and left overnight to ensure full chemical cross-linking before initiating in vitro release experiment.

### 5.3 Rheological Characterization of P407 Gel and Chemically Cross-Linked P407-10K Gels and P407-20K Gels

Rheological properties of the hydrogels were performed on a stress-controlled rheometer (ARES-G2, TA Instruments) using a parallel plate. For the P407 gel, 1 mL of 25% P407 polymer solution in 1× PBS was pipetted on the top of the rheometer plate. To quantify the gelation kinetics of the cross-linked hydrogels, 1 mL of 25 wt % P407-diacrylate polymer solution in 1× PBS was mixed with 50 mg of 8-arm-PEG10K-SH and then was pipetted on the top of the rheometer plate for the P407-10K gel and 1 mL of 25 wt % P407-diacrylate polymer solution in 1× PBS was mixed with 100 mg of 8-arm-PEG20K-SH and then pipetted on the top of the rheometer plate for P407-20K gel. The sample was covered with solvent traps to minimize water evaporation during the experiment. First at 4 °C and 37 °C, frequency sweep was performed at a range from 0.1 to 10 rad/s. Next, the temperature was increased from 4 °C to 37 °C and storage or elastic modulus (*G*′) and loss or viscous modulus (*G*″) were measured as a function time at angular frequencies of 6 rad/s and 3% strain amplitude chosen from the linear viscoelastic region. To measure the rheological properties for P407-10K gel and P407-20K gel, the gels were preformed at 37 °C using a 40 mm XRF Sample Cup (Premier lab supply, Lucie, FL) on the top of a polypropylene film. Using a parallel plate, the storage or elastic modulus (*G*′) and loss or viscous modulus (*G*″) were first measured via frequency sweep at 4 °C and 37 °C at a range from 0.1 to 10 rad/s. Then, elastic modulus (*G*′) and loss or viscous modulus (*G*″) were measured as a function time via temperature ramp at 4 °C and 37 °C using angular frequency of 6 rad/s and 3% strain amplitude chosen from the linear viscoelastic region.

### 5.4 Swelling Ratio Experiments

P407-10K gels and P407-20K gels (0.5 mL) as described above were incubated at 37 °C in 3 mL of 1× PBS for 70 days in triplicates. At predetermined time points (every 7 days), the mass of hydrogels after incubation was measured after removing and blotting off excess 1× PBS from the hydrogel. After measuring the mass of the hydrogel, fresh 3 mL of 1× PBS was added to continue the swelling experiment.

The equilibrium mass swelling ratio *q* was then determined using the following equation:^104^

$$q=\frac{W_{\mathrm{swollen}}}{W_{\mathrm{dry}}}$$

where *W*_dry_ is the average weight of the hydrogel determined after dried under vacuum overnight (*n* = 3) and *W*_swollen_ are the average weights (*n* = 3) of the swollen hydrogels at each specific time point.

### 5.5 In Vitro Protein Release Experiment

For the protein release experiments, P407-10K gels and P407-20K gels (0.5 mL) were prepared in triplicate in glass vials. 1× PBS release media (3 mL) was added over the formed hydrogels. Control samples of 40 mg/mL BSA and 20 mg/mL adalimumab in 1× PBS (not encapsulated in P407 hydrogel) were also prepared. All of the samples were then incubated in a 37 °C incubator (with orbital shaking at 50 rpm). At predetermined time points, the top layer release media with released cargo molecules was completely withdrawn and replenished with an equal amount (3 mL) of fresh PBS buffer. To quantify the concentration of the proteins, the withdrawn release media was diluted 2-fold with fresh 1× PBS. Then, 50 μL of the solution was mixed with 150 μL of Pierce 660 nm Protein Assay Reagent (Thermo Fisher, Waltham, MA). The absorbance at 660 nm was measured using a Spectramax M5 microplate reader (Molecular Devices, San Jose, CA). The concentration of the protein was determined based on the standard curves of known protein concentrations incorporating equal volume of fresh 1× PBS and release media from the cross-linked P407 hydrogel that did not encapsulate protein to minimize the signal interference. After the concentrations of release media at predetermined time points were determined, the cumulative release percentage (*P*) from the P407-10K gels and P407-20K gels was determined. Using the following equation:^42^

$$P=\frac{V_{e}\sum_{1}^{n-1}C_{i}+V_{0}C_{n}}{m_{\mathrm{protein}}}\times 100\%$$

where *m*_protein_ represents the amount of total protein (BSA: 40 mg or adalimumab: 20 mg) encapsulated in the hydrogel, *V*_0_ is the total volume of the release media (ranging from 2.5 to 3 mL at different time points), *V*_e_ is the volume of each sample that is being taken out at each time point (ranging from 2.5 to 3 mL at different time points), and *C*_*i*_ represents the concentration of the protein measured by the Pierce 660 nm protein assay in the *i*th sample where *C*_*n*_ represents the concentration of the *n*th sample. For the P407 gel (0.5 mL), the cumulative release of either BSA or adalimumab was calculated using the above equation where *m*_protein_ represents the amount of total protein (BSA: 40 mg or adalimumab: 20 mg) encapsulated in the hydrogel, *V*_0_ is the whole volume of the release media (3 mL), *V*_e_ is the volume of each sample that is being taken out at each time point (1 mL), and *C*_*i*_ represents the concentration of the protein measured by the Pierce 660 nm protein assay in the *i*th sample where *C*_*n*_ represents the concentration of the *n*th sample.

### 5.6 Sample Preparation for the Biophysical Characterization of Released Proteins

To minimize any signal interference coming from polymer fragments, release media of hydrogels from predetermined time points (day 1 through day 4, days 7, 14, 21, 28, 35, 42, 49, 56, 63, and 70, 3 mL in triplicates, total of 9 mL) were first concentrated using Amicon Ultra-15 mL 10 kDa molecular weight cutoff (MWCO) centrifugal filters (Burlington, MA Centrifugal Filters (Burlington, MA) to reduce the volume to less than 1 mL. For BSA, the concentrated release media was precipitated using 1000 μL of ice-cold acetone that contains 300 mM NaCl. Then, the samples were centrifuged at 14,000 × *g* for 10 min at 4 °C. The supernatant was decanted, and the pellet was washed with ice-cold acetone twice more; the pellet was then dissolved in 100 μL of nuclease-free water (AM9932, Thermo Fisher, Waltham, MA). The concentration of BSA was determined by absorption at 280 nm (mass extinction coefficient of 6.7 mL·mg^−1^·cm^−1^) using a NanoDrop 8000 (Thermo Fisher, Waltham, MA). For adalimumab purification, the concentrated release media was run through Nab Protein A Plus Spin Kit (Thermo Fisher, Waltham, MA), following the manufacturer’s protocol. The purified adalimumab was then buffer exchanged with nuclease-free water using Amicon Ultra-0.5 mL of 10 kDa MWCO centrifugal filters (Burlington, MA) at a volume of 100 μL. The concentration of adalimumab was then determined by absorption at 280 nm (mass extinction coefficient of 1.39 mL·mg^−1^·cm^−1^) using NanoDrop 8000 (Thermo Fisher, Waltham, MA) for downstream biophysical characterizations.

### 5.7 Size-Exclusion Chromatography

Size-exclusion chromatography (SEC) studies were performed on an Acquity H-Class UPLC (Waters, Milford, MA) with a protein BEH SEC 200 Å column (1.7 μm, 4.6 mm × 150 mm, 186005225). In a typical experiment, 2 mg/mL of 10 μL of protein sample was injected into a UPLC and eluted with either 1× PBS (BSA) or 50 mM pH 7.0 phosphate buffer containing 200 mM potassium chloride (adalimumab) at a flow rate of 0.25 mL/min. The detection of protein samples was performed with a UV detector (Waters UV/visible detector 2489) at 280 nm. The chromatogram was analyzed with Empower 3 Chromatography Data Software.

### 5.8 Circular Dichroism

CD spectroscopy of the released protein samples was recorded on a JASCO J-715 spectrophotometer. To record the spectra, 400 μL of 1 mg/mL sample solution in nuclease-free water was pipetted in a quartz cuvette of 1 mm path length and scanned from 200 to 260 nm at 25 °C (scan rate: 10 nm/min, bandwidth: 1 nm, data pitch: 0.05 nm). The measurements were taken in triplicates, and the average values were plotted as mean residue ellipticity ([θ]_MRE_) using the following equation:^105^

$$[\theta {]}_{\mathrm{MRE}}=\frac{\mathrm{MRW}\times \theta_{\mathrm{obs}}}{c\times l\times N}$$

where MRW is the mean residue weight, which is typically 100 Da for proteins, θ_obs_ is the observed ellipticity in the CD spectrometer, *c* is the concentration of the protein solution in mg/mL, *l* is the path length of the cuvette in cm, and *N* is the number of amino acid residues in the protein.

### 5.9 Capillary Electrophoresis Sodium Dodecyl Sulfate (CE-SDS)

CE-SDS experiments were performed using a Maurice CE-SDS application kit from ProteinSimple (Bio-Techne, San Jose, CA, USA), containing CE-SDS cartridges, vials, caps, and 96-well plates, as well as CE-SDS separation matrix, sample buffer, wash and conditioning solutions, and internal standards. To prepare samples for the CE-SDS, the released proteins that were either precipitated (BSA) or purified by Nab Protein A Plus Spin Kit (adalimumab) were diluted with nuclease free water to 2 mg/mL concentration at a volume of 25 μL. Then, 25 μL of Maurice 1× Sample buffer was added along with 2 μL of Maurice CE-SDS 25× Internal Standard (10 kDa recombinant protein). For reduced CE-SDS, 2.5 μL of 14.2 M β-mercaptoethanol (Sigma-Aldrich, St. Louis, MO) stock solution was added. For nonreduced CE-SDS, 2.5 μL of 250 mM iodoacetamide (Sigma-Aldrich, St. Louis, MO) stock solution was added to the samples to prevent disulfide scrambling or exchange. The samples were then vortexed and heated to 70 °C for 10 min. After cooling down on ice, the samples were vortexed again and were centrifuged using a mini centrifuge to remove bubbles in the sample. Then, 50 μL of each sample was loaded into a 96-well plate. To run the CE-SDS, the 96-well plate was loaded onto a Maurice (Bio-Techne, San Jose, CA). All samples were electrokinetically injected into the cartridge capillary by applying 4600 V for 20 s and sample separation by electrophoresis at 5750 V for 35 min for reduced CE-SDS and 45 min for nonreduced CE-SDS. The data were evaluated by Compass for iCE 4.0.0 (Bio-Techne, San Jose, CA, USA) to analyze the relative migration time (RMT) relative to the CE-SDS 25× Internal Standard 10 kDa (recombinant protein).

### 5.10 Adalimumab Bioactivity Assay

Adalimumab samples collected released from the hydrogels at days 63 and 70 were analyzed using TNF-alpha: TNF receptor 1 inhibitor screening enzyme-linked immunosorbent assay (ELISA) kit (ACRO Biosystems, Newark, DE) according to the manufacturer’s protocol. Fresh stock of adalimumab (stored at −80 °C) and 20 mg/mL adalimumab solution in 1× PBS incubated for 70 days at 37 °C were used as control groups. The half maximal inhibitory concentration (IC50) was determined using GraphPad Prism 10.2.2’s (Graphpad Software LLC, San Diego, USA) built-in model: [inhibitor] vs response-variable slope (four parameters) analysis. All data points were collected in triplicates.

### 5.11 Statistical Analysis

All the results with 95% confidence band were plotted using Graphpad Prism 10.2.2 (Graphpad Software LLC, San Diego, USA) with nonlinear regression fitting. For comparisons of the IC50 values in ELISA assay, means were assessed using an ordinary one-way analysis of variance (ANOVA) followed by Tukey’s multiple comparison test (sample size n = 3). *p* values of less than 0.05 were considered statistically significant. For time-dependent measurement results, to account for the correlation within the samples, repeated measure analysis was conducted using JMP statistical software (SAS institute, Cary, NC), where the samples (sample size *n* = 3) were incorporated as a random effect; time, hydrogel system, and the interaction between time and hydrogel system were chosen as parameters. Followed by repeated measure analysis, a two-tailed *t* test was used to determine if there is a statistically significant difference between the means of two groups, and *p* values less than 0.05 were considered statistically significant. For both statistical analysis, normal distribution was assumed, as is a common practice in related literature.

## Supplementary Material



## Notes

Method for calculating the mesh size of the cross-linked P407 hydrogels; rheometry plots describing the gelation kinetics of the cross-linked P407-10K gel and P407-20K gel; the experimental method for quantifying unreacted 8-arm-PEG-SH linkers and free sulfhydryl groups in BSA; SEC spectra of the release media from P407-10K gel and P407-20K gel; SEC spectra of BSA and adalimumab released from cross-linked P407 hydrogels; reduced CE-SDS electropherograms of BSA and adalimumab released from P407-10K gels and P407-20K gels; nonreduced CE-SDS electropherograms of adalimumab released from P407-10K gels and P407-20K gels; the release kinetics of human serum IgG from P407-10K gels and P407-20K gels; proposed target product profile of cross-linked hydrogels; results of the repeated measure analysis for the swelling ratio, release kinetics of BSA, adalimumab, and human serum IgG; results of calculated mesh sizes and cross-linking density of P407, P407-10K, and P407-20K gels; ordinary one-way ANOVA analysis followed by Tukey’s multiple comparison test to compare the IC50 values of adalimumab (PDF)

## #

Bioproduct Research & Development, Lilly Technology Center-North, Indianapolis, Indiana, 46221, United States
