# Supplementary material for: Engineering Extended Release Profiles for Biologic Formulations via Chemical Cross-Linking of Poloxamer 407 Hydrogels
Source: ACS Omega. 2025 Nov 24;10(48):58147–63. doi: 10.1021/acsomega.5c02571 (PMC12771245; doi:10.1021/acsomega.5c02571)
Supplement: Supplementary file 1 [file ao5c02571_si_001.pdf]

Supporting information

Engineering extended release profiles for biologic formulations via chemical crosslinking of Poloxamer 407 hydrogels.

Jungsoo Park [1], *Yu-Jiun Lin* [2], *Kingshuk Dutta* [1 #], *Seth Forster* [1], *Grace Okoh* [1], *Yu Tian* [2] *and Yingkai Liang* [*1]

[1] Discovery Pharmaceutical Sciences, Merck & Co., Inc., West Point, Pennsylvania, 19486, United States

[2] Materials and Biophysical Characterization, Merck & Co., Inc., Rahway, New Jersey, 07065, United States

Present address:

K.D: Bioproduct Research & Development, Lilly Technology Center-North, Indianapolis, Indiana, 46221, United States

*Address correspondence to: yu.tian9@merck.com & yingkai.liang@merck.com

Ellman's reagent assay to detect unreacted 8-arm-PEG-SH crosslinkers from chemically crosslinked P407 hydrogels.

The hydrogels were formulated by Michael-type addition between 8-arm PEG-SH and P407-diacrylate polymer. At 4°C, 0.5 mL of 25 weight percent (wt %) P407-diacrylate polymer solution was first aliquoted to a 4 mL glass vial at 4°C. For P407-10K gel and P407-20K gel, 25 mg of 8-arm-PEG10K-SH and 50 mg of 8-arm-PEG20K-SH was added to the dissolved polymer solution, respectively. For both gels, the molar ratio between the thiols and the acrylates was 1:1. After confirming the PEG crosslinkers were fully dissolved within the polymer solution, the vials were then transferred to 37°C incubator and were left over night to achieve maximum cross-linking. After overnight incubation, 2.5 mL of 1× PBS (pH 10.0) was then added to the glass vials and were incubated at 37°C for 3 days to disintegrate the gel. Disintegrated gel solution then was buffer exchanged with 1× PBS (pH 7.4) using Amicon 10K molecular weight cut off (MWCO) 5 mL spin filter. 50 µL of solution was then added to the 96 well plate well as well as the standard curve was generated by adding 50 µL of 8-arm-PEG10K-SH and 8-arm-PEG20K-SH dissolved in 1× PBS ranging from (0 mg/mL to 2.5 mg/mL). Then freshly prepared Ellman's reagent (Thermo Fisher, 3 mg/mL solution, dissolved in 0.1 M pH 8.0 phosphate buffer) was added to each well at a volume of 50 µL. After stirring the plate for 1 minute, the absorbance at 412 nm was recorded using the measured using the Spectramax M5 microplate reader (Molecular Devices, San Jose, CA). Then, the concentration of 8-arm-PEG-SH was determined based on the standard curve to determine the percentage of the unreacted 8-arm-PEG-SH cross-linkers.

Ellman's reagent assay to detect concentration of free sulfhydryl groups in BSA extracted from P407-10K gels and P407-20K gels.

8-arm-PEG-SH cross-linkers were first added to the polymer solution as mentioned above. After the PEG cross-linkers were fully dissolved, 20 mg of lyophilized BSA powder (Sigma Aldrich, St. Louis, MO) was directly added to the polymer and cross-linker mixture to achieve a final concentration of 40 mg/mL at 4°C. After confirming the BSA was fully dissolved in the polymer and cross-linker mixture, the vials were incubated at 37°C. After overnight incubation, 2.5 mL of 1× PBS (pH 10.0) was added to the hydrogels to completely disintegrate the hydrogel and were incubated at 37°C for 3 days. Then, 3.0 mL of ice-cold acetone that contains 300 mM NaCl was added to the vial to precipitate out the encapsulated BSA. Samples were then centrifuged at 14,000 ×g for 10 minutes at 4°C. The supernatant was decanted and was washed with ice-cold acetone twice more and the pellet was resuspended in 1× PBS (pH 7.4). The concentration of BSA was determined by absorption at 280 nm (extinction coefficient of 6.7) using NanoDrop 8000 (Thermo Fisher, Waltham, MA). Then in a 96 well plate, 50 μL of BSA solution (90.4 μM, 6 mg/mL) was added to the well as well as the standard curve was created by adding 50 μL of freshly prepared BSA solution in 1× PBS (pH 7.4) ranging from (0 mg/mL to 10 mg/mL). Then freshly prepared Ellman's reagent (Thermo Fisher, 3 mg/mL solution, dissolved in 0.1 M pH 8.0 phosphate buffer) was added to each well at a volume of 50 μL. After stirring the plate for 1 minute, the absorbance at 412 nm was recorded using the measured using the Spectramax M5 microplate reader (Molecular Devices, San Jose, CA). The percentage of reacted sulfhydryl group in BSA extracted from gels (6 mg/mL) was determined by comparing the concentration of free sulfhydryl group in freshly prepared BSA (6 mg/mL) in 1X PBS (pH 7.4).

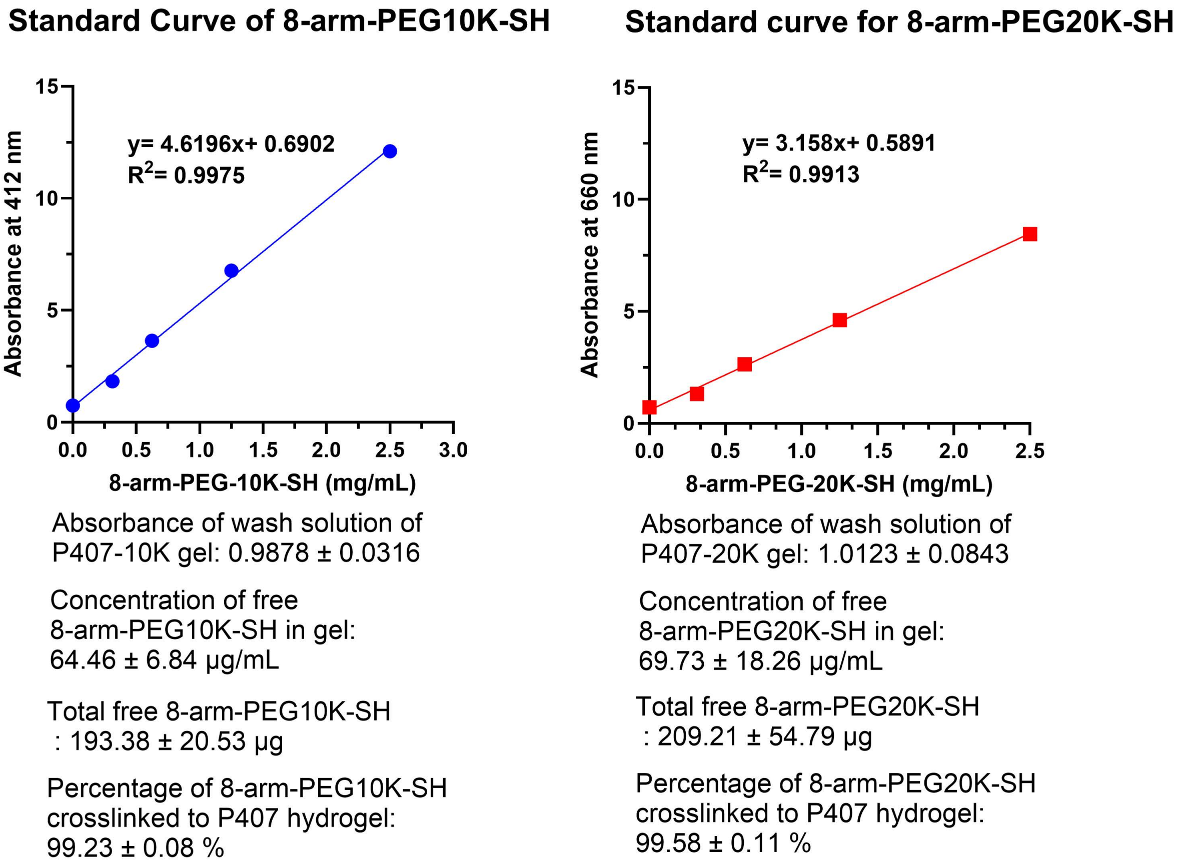

Figure S1. Quantification of unreacted 8-arm-PEG10K-SH and 8-arm-PEG20K-SH from P407-10K gel and P407-20K gel, respectively, was assessed using Ellman's reagent. Standard curves were created based on known concentrations of 8-arm-PEG10K-SH and 8-arm-PEG20K-SH. Using these standard curves, the concentrations of unreacted 8-arm-PEG10K-SH and 8-arm-PEG20K-SH in P407-10K and P407-20K were interpolated, respectively. From these data, the percentage of 8-arm-PEG10K-SH and 8-arm-PEG20K-SH crosslinked to P407-10K gel and P407-20K gel, respectively, was determined.

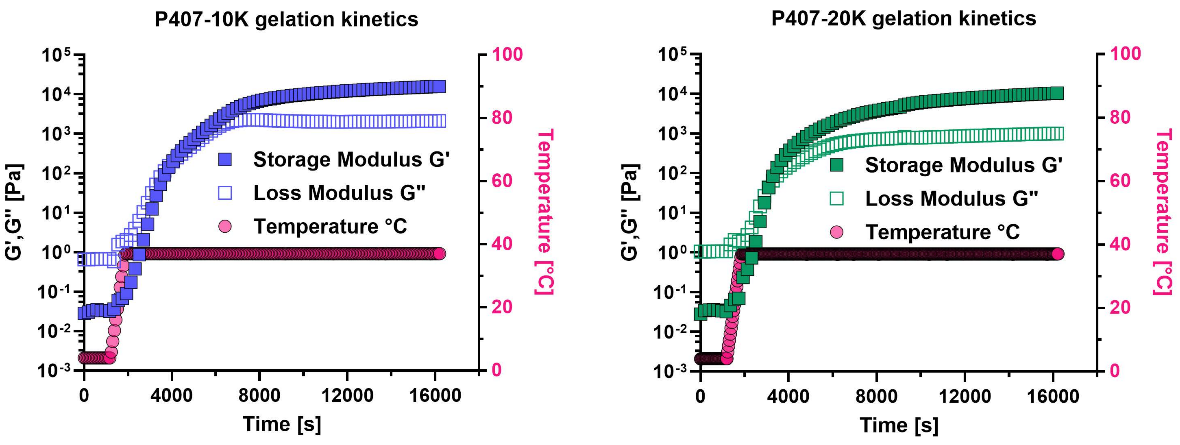

Figure S2. *In situ* rheological characterization of P407-10K gel (P407-diacrylate chemically crosslinked with 8-arm-PEG10K-SH) and P407-20K gel (P407-diacrylate chemically crosslinked with 8-arm-PEG20K-SH) during gelation process as a function of time via temperature sweep at 6 rad/s and 3% strain.

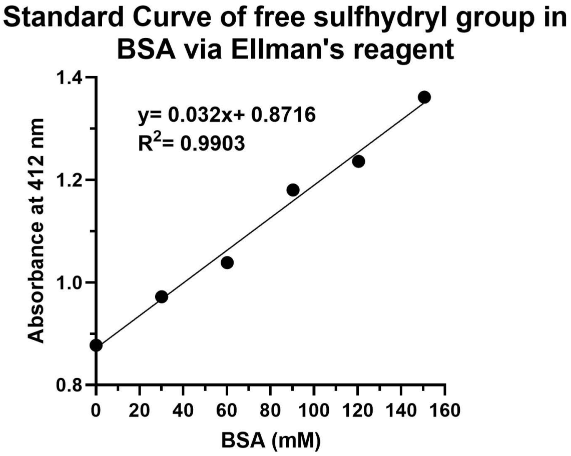

Absorbance of freshly prepared 90.4 μM BSA (6 mg/mL): 1.159 ± 0.004

Concentration of free sulfhydryl group in 90.4 μM BSA(6 mg/mL): 89.92 ± 1.10 μM

Absorbance of 90.4 μM BSA (6 mg/mL) extracted from P407-10K gel: 1.059 ± 0.016

Concentration of free sulfhydryl group in 90.4 μM BSA (6 mg/mL) extracted from P407-10K gel: 58.66 ± 4.94 μM

Percentage of BSA reacted with polymers : 34.72 ± 5.56%

Absorbance of 90.4 μM BSA (6 mg/mL) extracted from P407-20K gel: 0.969 ± 0.016

Concentration of free sulfhydryl group in 90.4 μM BSA (6 mg/mL) extracted from P407-20K gel: 30.33 ± 5.29 μM

Percentage of BSA reacted with polymers : 66.13 ± 6.05%

Figure S3. Quantification of free sulfhydryl group in BSA extracted from P407-10K gel and P407-gel. A standard curve was generated using known freshly prepared BSA concentrations as a function of absorbance at 412 nm. Based on the standard curves, concentration of free sulfhydryl group in BSA extracted from P407-10K gel and P407-20K gel was calculated to determine the fraction of BSA that is bound to P407-diacrylate polymer.

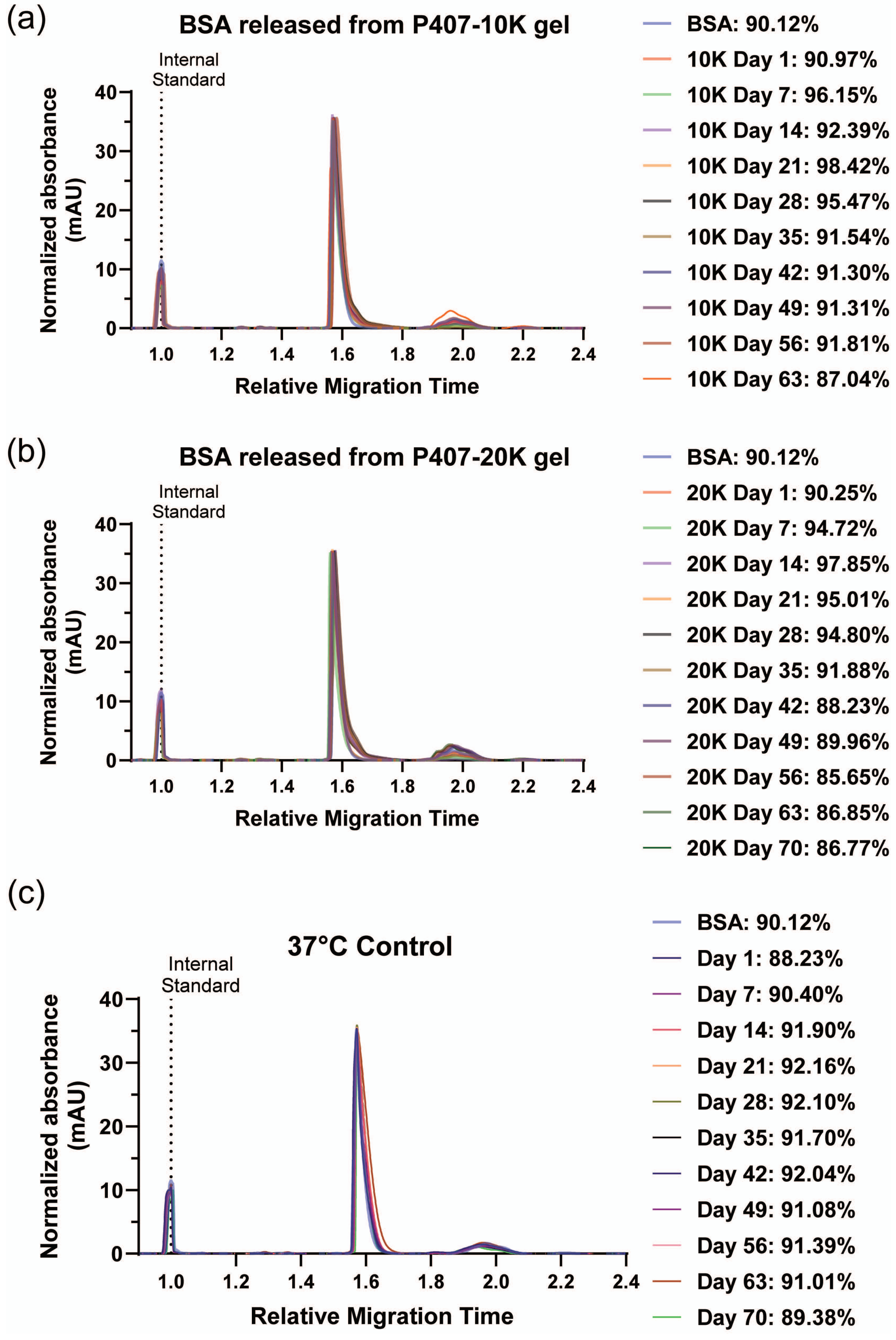

Figure S4. Electropherogram from reduced CE-SDS for BSA released from (a) P407-10K gel, (b) P407-20K gel and (c) BSA incubated at 40 mg/mL in pH 7.4, 1× PBS at 37°C at different time points during the 70-day release/incubation period. The percentage of monomers at respective time points are listed in the legend. (n=1)

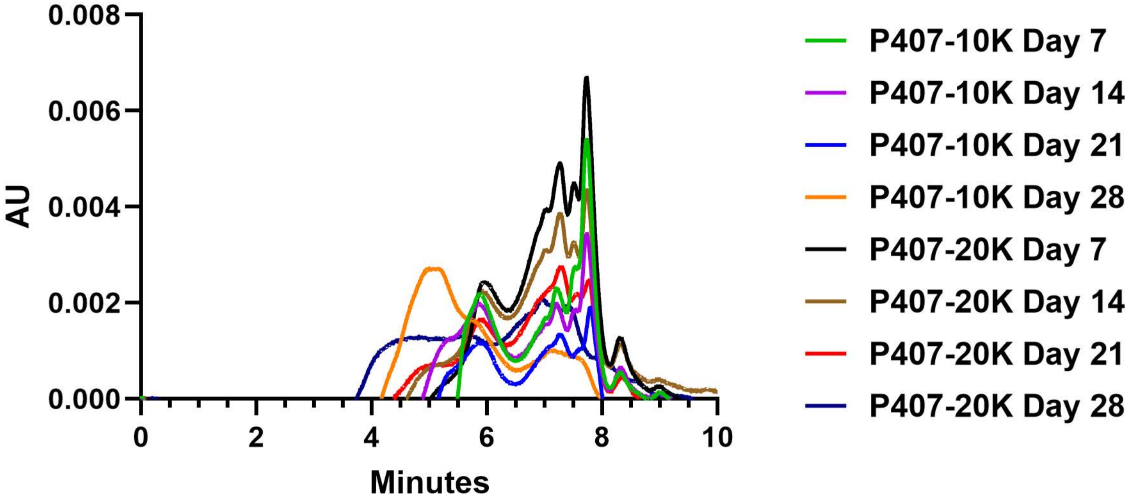

Figure S5. SEC chromatograms of release media from chemically crosslinked hydrogels, which did not contain biologics, were incubated in pH 7.4, 1× PBS at 37°C for various durations, as specified. Heterogenous hydrogel fragments in the release media from the chemically crosslinked P407 hydrogels were detected.

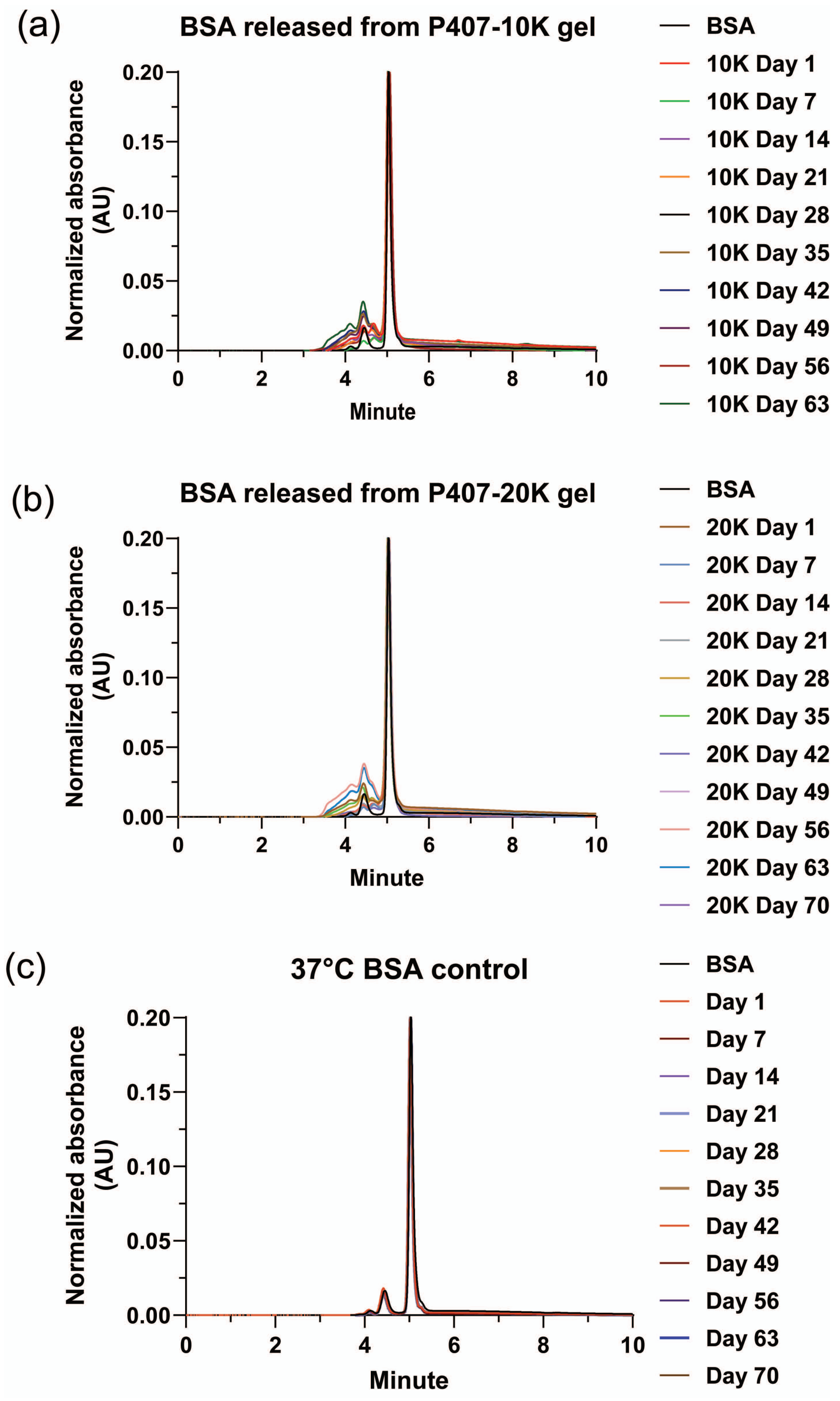

Figure S6. SEC spectra of BSA released from (a) P407-10K gel, (b) P407-20K gel and (c) BSA incubated at 40 mg/mL in pH 7.4, 1× PBS at 37°C at different time points during the 70-day release/incubation period. (n=1)

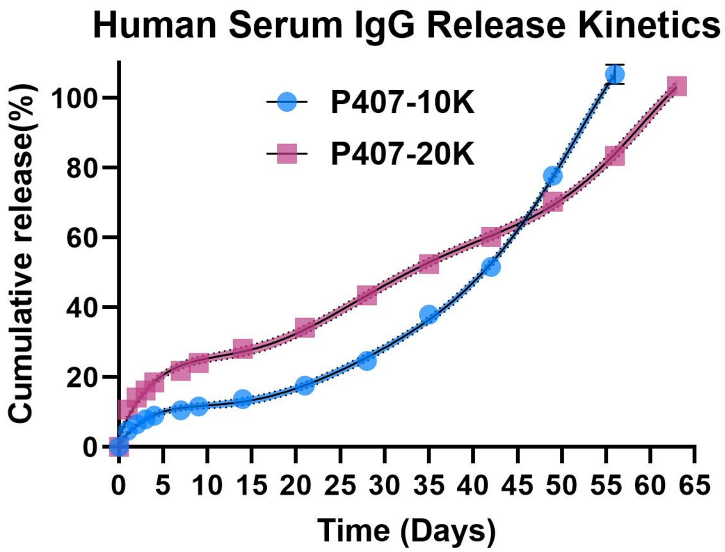

Figure S7. Cumulative release of human serum IgG from P407-10K and P407-20K hydrogel formulations in 1×PBS, pH 7.4 at 37°C (average ± S.D, n=3), with 95% confidence band. Repeated measures analysis revealed that the cumulative release percentage of human serum IgG between P407-10K gel and P407-20K gel is statistically significantly different from one another, except for days 1 and day 2. ($p < 0.05$)

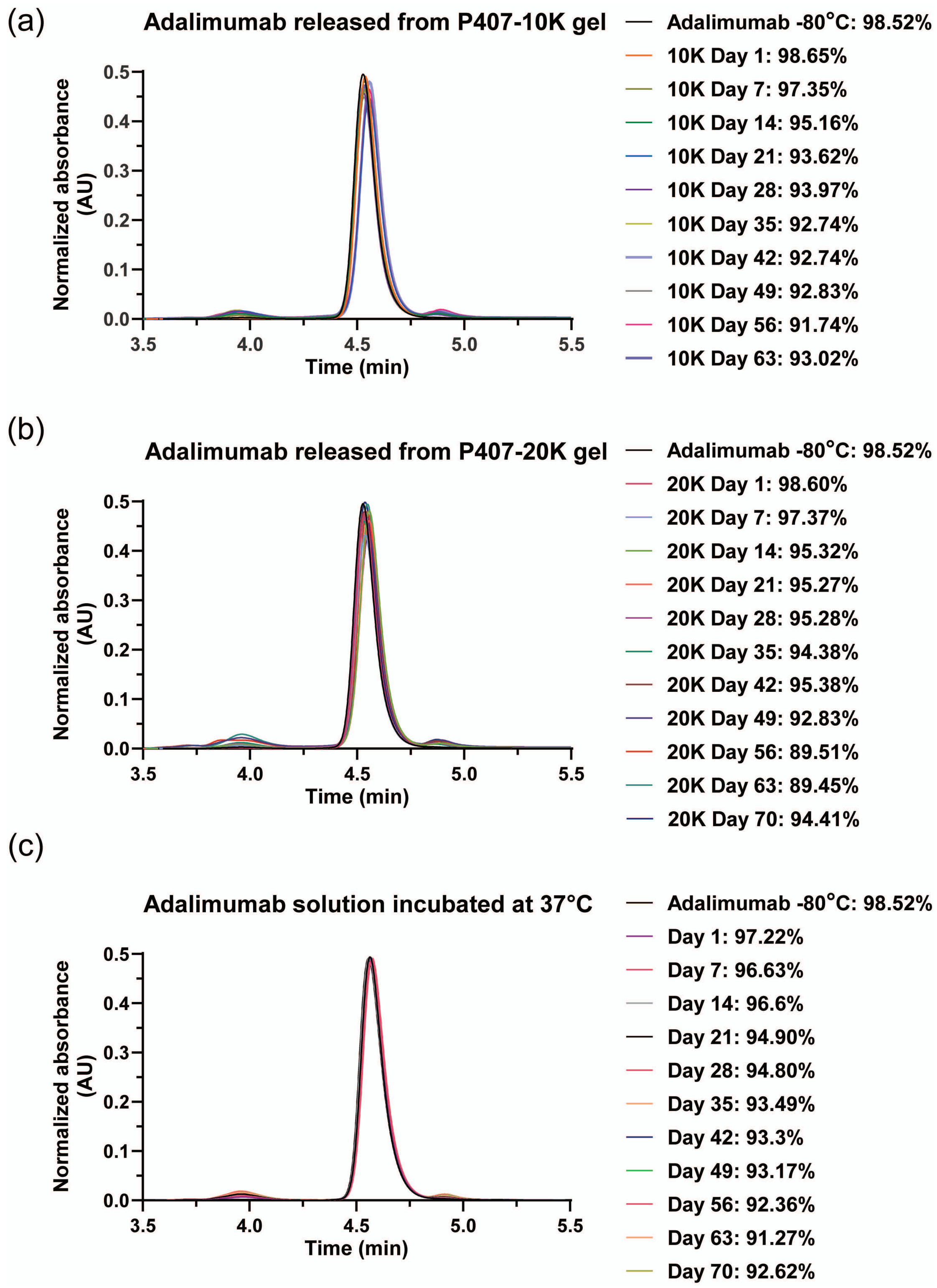

Figure S8. SEC spectra of adalimumab released from (a) P407-10K gel, (b) P407-20K gel and (c) adalimumab incubated at 20 mg/mL in pH 7.4, 1× PBS at 37°C at different time points during the 70-day release/incubation period. The percentage of monomer fraction at respective time points are listed in the legend. (n=1)

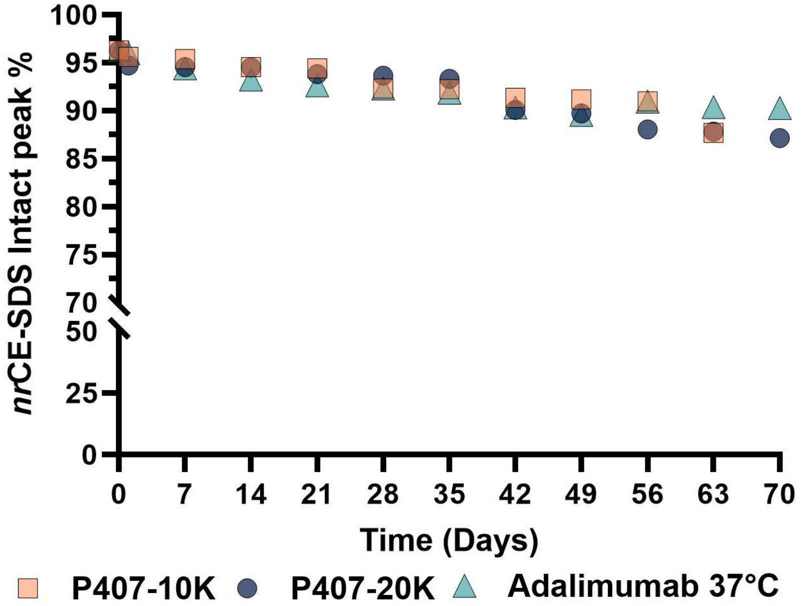

Figure S9. Scatter plot of the intact peak percentage of adalimumab in non-reduced CE-SDS from P407-10K gel, P407-20K gel and adalimumab incubated at 20 mg/mL in pH 7.4, 1× PBS at 37°C at different time points during the 70-day release/incubation period. (n=1)

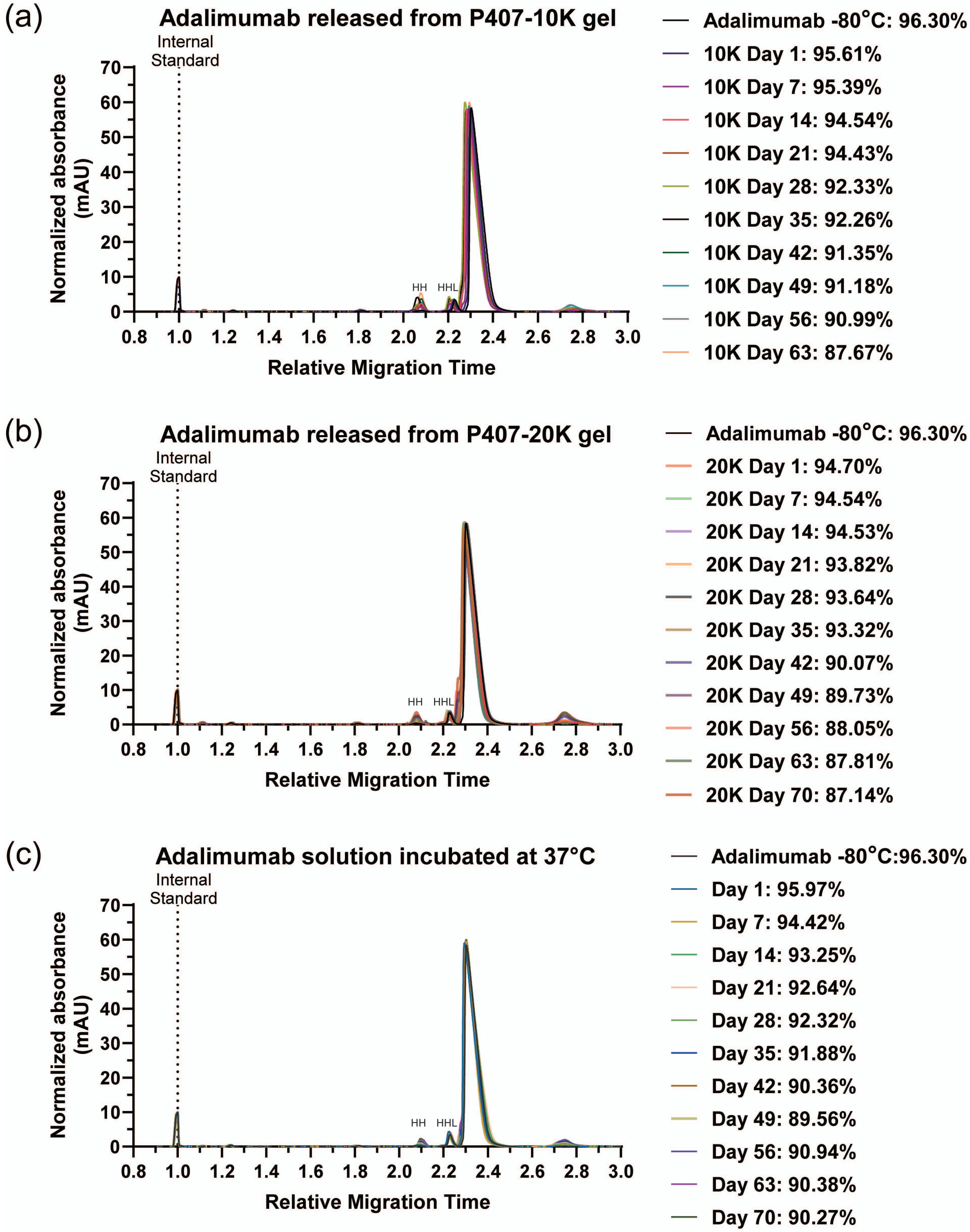

Figure S10. Non-reduced CE-SDS electropherogram of adalimumab released from (a) P407-10K gel, (b) P407-20K gel and (c) adalimumab incubated at 20 mg/mL in pH 7.4, 1× PBS at 37°C at different time points during the 70-day release/incubation period. The percentage of monomer fraction at respective time points are listed in the legend. HH and HHL denotes heavy-heavy chain species and heavy-heavy-light chain species, respectively. (n=1)

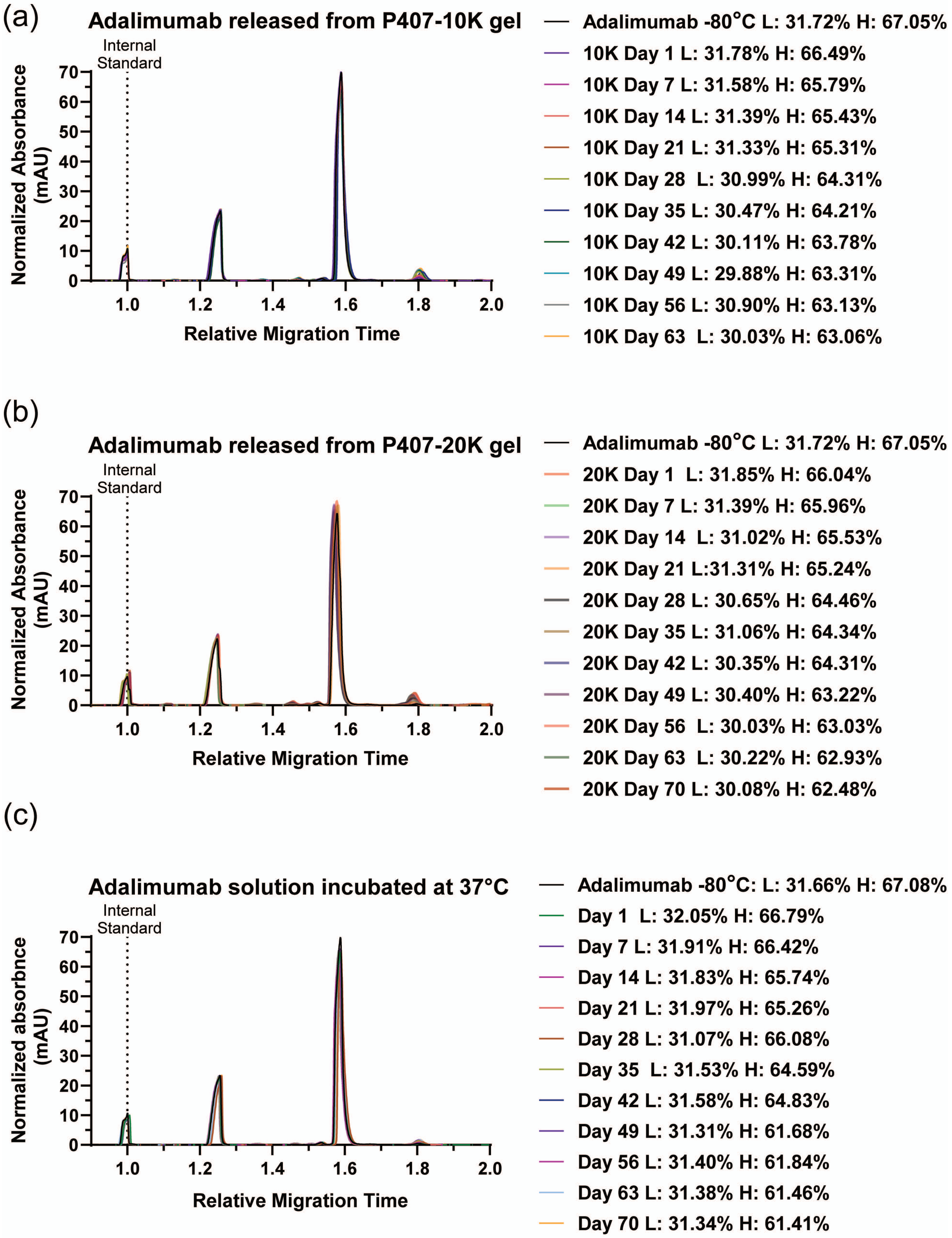

Figure S11. Reduced CE-SDS electropherogram of adalimumab released from (a) P407-10K gel, (b) P407-20K gel and (c) adalimumab incubated at 20 mg/mL in pH 7.4, 1× PBS at 37°C at different time points during the 70-day release/incubation period. The percentage of heavy chain and light chain at respective time points are listed in the legend. (n=1)

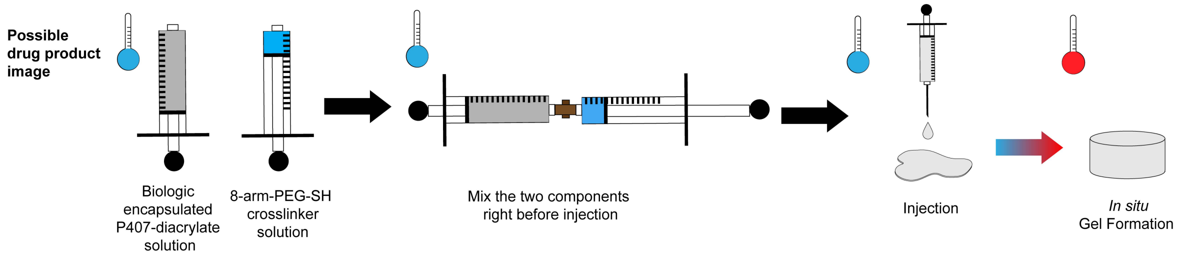

Figure S12. Proposed target product profile of crosslinked P407 hydrogel. Biologics can be encapsulated in a P407 diacrylate-polymer solution in one syringe and chemical crosslinkers in another syringe. This setup would allow for cold chain logistics during transport, with the components mixed immediately at ambient temperature before injection and then form an *in situ* hydrogel.

Response Swelling ratio
Effect Summary

Summary of Fit

RSquare	0.997144
RSquare Adj	0.995776
Root Mean Square Error	0.10462
Mean of Response	6.506189
Observations (or Sum Wgts)	72

Parameter Estimates

Term	Estimate	Std Error	DFDen	t Ratio	Prob>|t|	Lower 95%	Upper 95%
Intercept	6.5061885	0.16916	4	38.46	<.0001*	6.0365257	6.9758513
System[10K]	-0.765802	0.16916	4	-4.53	0.0106*	-1.235465	-0.29614
Time (Day)[0]	-3.35617	0.040892	44	-82.07	<.0001*	-3.438583	-3.273757
Time (Day)[1]	-1.384656	0.040892	44	-33.86	<.0001*	-1.467069	-1.302243
Time (Day)[7]	-0.499766	0.040892	44	-12.22	<.0001*	-0.58218	-0.417353
Time (Day)[14]	-0.125988	0.040892	44	-3.08	0.0036*	-0.208402	-0.043575
Time (Day)[21]	0.0788103	0.040892	44	1.93	0.0604	-0.003603	0.1612237
Time (Day)[28]	0.2208575	0.040892	44	5.40	<.0001*	0.1384441	0.3032709
Time (Day)[35]	0.3580295	0.040892	44	8.76	<.0001*	0.2756161	0.4404429
Time (Day)[42]	0.5355778	0.040892	44	13.10	<.0001*	0.4531644	0.6179912
Time (Day)[49]	0.7195407	0.040892	44	17.60	<.0001*	0.6371273	0.8019541
Time (Day)[56]	0.8939277	0.040892	44	21.86	<.0001*	0.8115143	0.9763411
Time (Day)[63]	1.1583995	0.040892	44	28.33	<.0001*	1.0759861	1.2408129
Time (Day)[0]*System[10K]	0.8177333	0.040892	44	20.00	<.0001*	0.7353199	0.9001467
Time (Day)[1]*System[10K]	0.3237806	0.040892	44	7.92	<.0001*	0.2413672	0.406194
Time (Day)[7]*System[10K]	0.1258804	0.040892	44	3.08	0.0036*	0.0434671	0.2082938
Time (Day)[14]*System[10K]	0.0213683	0.040892	44	0.52	0.6039	-0.061045	0.1037817
Time (Day)[21]*System[10K]	-0.042224	0.040892	44	-1.03	0.3074	-0.124638	0.040189
Time (Day)[28]*System[10K]	-0.069131	0.040892	44	-1.69	0.0980	-0.151545	0.0132822
Time (Day)[35]*System[10K]	-0.071217	0.040892	44	-1.74	0.0886	-0.15363	0.0111965
Time (Day)[42]*System[10K]	-0.115266	0.040892	44	-2.82	0.0072*	-0.197679	-0.032853
Time (Day)[49]*System[10K]	-0.134677	0.040892	44	-3.29	0.0020*	-0.217091	-0.052264
Time (Day)[56]*System[10K]	-0.20911	0.040892	44	-5.11	<.0001*	-0.291523	-0.126696
Time (Day)[63]*System[10K]	-0.287045	0.040892	44	-7.02	<.0001*	-0.369458	-0.204632

Table S1. Repeated measure analysis was conducted to assess the significant difference in the swelling ratio between P407-10K and P407-20K gels: In the model, time, system was taken into account to assess the system impact. Samples were incorporated as a random effect. Except for day 14 to day 35, the swelling ratio between P407-10K and P407-20K gels were significantly different from each other. Prob>|t| denotes the *p* value for the two-tailed test. p <0.05 was considered significantly different from each other.

	P407 Gel	P407-10K Gel	P407-20K Gel
**G′(Pa) at 0.1 rad/s**	6409.0	13362.8	10921.4
**G′(Pa) at 10 rad/s**	14126.6	18334.3	16514.9
**Mesh size at 37°C (nanometers: nm)**	6.7 - 8.7 nm	6.2 - 6.8 nm	6.4 -7.3 nm
**Crosslinking density at 37°C**	2.5 – 5.5 mol·m^{-3}	5.2 – 7.0 mol·m^{-3}	4.2 – 6.4 mol·m^{-3}

Table S2. Table of calculated of mesh sizes and crosslinking density of P407, P407-10K, P407-20K gels at a range from 0.1 to 10 rad/s at 37°C. Based on the storage modulus G′ from oscillatory rheology, The mesh size was calculated using the following equation[1, 2]:

$$\xi = \left(\frac{G' N_A}{RT}\right)^{-1/3}$$

Calculation of the crosslinking density n_e (mol·m^{-3}) of P407, P407-10K, P407-20K at a range from 0.1 to 10 rad/s was calculated using the following equation[2, 3]:

$$n_e = \frac{G'}{RT}$$

Where G′ is the storage modulus determined at 0.1 rad/s and 10 rad/s, respectively, N_A is the Avogadro's number (6.022 ×10^{23}), R is the gas constant (8.314 J/K mol), and T is the temperature at 37°C which is 310K.

Response BSA Cumulative Release (%)
Effect Summary
Summary of Fit

RSquare	0.999842
RSquare Adj	0.999766
Root Mean Square Error	0.444036
Mean of Response	27.68444
Observations (or Sum Wgts)	90

Parameter Estimates

Term	Estimate	Std Error	DFDen	t Ratio	Prob>|t|
Intercept	27.684439	0.047715	4	580.21	<.0001*
Time (day)[0]	-27.68444	0.17513	56	-158.1	<.0001*
Time (day)[1]	-17.76634	0.17513	56	-101.4	<.0001*
Time (day)[2]	-17.59933	0.17513	56	-100.5	<.0001*
Time (day)[3]	-17.45679	0.17513	56	-99.68	<.0001*
Time (day)[4]	-17.29919	0.17513	56	-98.78	<.0001*
Time (day)[7]	-16.88108	0.17513	56	-96.39	<.0001*
Time (day)[14]	-15.28698	0.17513	56	-87.29	<.0001*
Time (day)[21]	-13.37707	0.17513	56	-76.38	<.0001*
Time (day)[28]	-10.15639	0.17513	56	-57.99	<.0001*
Time (day)[35]	-5.857293	0.17513	56	-33.45	<.0001*
Time (day)[42]	-0.266076	0.17513	56	-1.52	0.1343
Time (day)[49]	7.4435473	0.17513	56	42.50	<.0001*
Time (day)[56]	19.987416	0.17513	56	114.13	<.0001*
Time (day)[63]	59.043642	0.17513	56	337.14	<.0001*
System[P407-10K]	-2.007938	0.047715	4	-42.08	<.0001*
Time (day)[0]*System[P407-10K]	2.0079375	0.17513	56	11.47	<.0001*
Time (day)[1]*System[P407-10K]	-1.172425	0.17513	56	-6.69	<.0001*
Time (day)[2]*System[P407-10K]	-1.245258	0.17513	56	-7.11	<.0001*
Time (day)[3]*System[P407-10K]	-1.248716	0.17513	56	-7.13	<.0001*
Time (day)[4]*System[P407-10K]	-1.213407	0.17513	56	-6.93	<.0001*
Time (day)[7]*System[P407-10K]	-1.20339	0.17513	56	-6.87	<.0001*
Time (day)[14]*System[P407-10K]	-1.064195	0.17513	56	-6.08	<.0001*
Time (day)[21]*System[P407-10K]	-1.742594	0.17513	56	-9.95	<.0001*
Time (day)[28]*System[P407-10K]	-2.553871	0.17513	56	-14.58	<.0001*
Time (day)[35]*System[P407-10K]	-3.426756	0.17513	56	-19.57	<.0001*
Time (day)[42]*System[P407-10K]	-4.003296	0.17513	56	-22.86	<.0001*
Time (day)[49]*System[P407-10K]	-1.763132	0.17513	56	-10.07	<.0001*
Time (day)[56]*System[P407-10K]	2.6789892	0.17513	56	15.30	<.0001*
Time (day)[63]*System[P407-10K]	14.783009	0.17513	56	84.41	<.0001*

Table S3. Repeated measure analysis was conducted to assess the significant difference in the BSA cumulative release between P407-10K and P407-20K gels: In the model, time, system was taken into account to assess the system impact. Samples were incorporated as a random effect. Except for day 42, BSA cumulative release between P407-10K and P407-20K gels were significantly different from each other. Prob>|t| denotes the p value for the two-tailed test. $p < 0.05$ was considered significantly different from each other.

Response adalimumab Cumulative release (%)
Effect Summary
Summary of Fit

RSquare	0.995403
RSquare Adj	0.993181
Root Mean Square Error	2.758133
Mean of Response	40.52756
Observations (or Sum Wgts)	90

Parameter Estimates

Term	Estimate	Std Error	DFDen	t Ratio	Prob>|t|
Intercept	40.527559	0.302958	4	133.77	<.0001*
Time (day)[0]	-40.52756	1.087822	56	-37.26	<.0001*
Time (day)[1]	-30.84011	1.087822	56	-28.35	<.0001*
Time (day)[2]	-27.19622	1.087822	56	-25.00	<.0001*
Time (day)[3]	-25.25636	1.087822	56	-23.22	<.0001*
Time (day)[4]	-24.16936	1.087822	56	-22.22	<.0001*
Time (day)[7]	-22.88993	1.087822	56	-21.04	<.0001*
Time (day)[14]	-20.13576	1.087822	56	-18.51	<.0001*
Time (day)[21]	-17.76011	1.087822	56	-16.33	<.0001*
Time (day)[28]	-6.88793	1.087822	56	-6.33	<.0001*
Time (day)[35]	4.6336048	1.087822	56	4.26	<.0001*
Time (day)[42]	15.67735	1.087822	56	14.41	<.0001*
Time (day)[49]	32.45679	1.087822	56	29.84	<.0001*
Time (day)[56]	47.270258	1.087822	56	43.45	<.0001*
Time (day)[63]	56.067639	1.087822	56	51.54	<.0001*
System[P407-10K]	-1.866352	0.302958	4	-6.16	0.0035*
Time (day)[0]*System[P407-10K]	1.8663516	1.087822	56	1.72	0.0917
Time (day)[1]*System[P407-10K]	0.3404307	1.087822	56	0.31	0.7555
Time (day)[2]*System[P407-10K]	-0.015907	1.087822	56	-0.01	0.9884
Time (day)[3]*System[P407-10K]	-0.544847	1.087822	56	-0.50	0.6184
Time (day)[4]*System[P407-10K]	-1.116177	1.087822	56	-1.03	0.3093
Time (day)[7]*System[P407-10K]	-2.25152	1.087822	56	-2.07	0.0431*
Time (day)[14]*System[P407-10K]	-4.631487	1.087822	56	-4.26	<.0001*
Time (day)[21]*System[P407-10K]	-5.991695	1.087822	56	-5.51	<.0001*
Time (day)[28]*System[P407-10K]	-4.84056	1.087822	56	-4.45	<.0001*
Time (day)[35]*System[P407-10K]	-3.334615	1.087822	56	-3.07	0.0033*
Time (day)[42]*System[P407-10K]	-0.50999	1.087822	56	-0.47	0.6410
Time (day)[49]*System[P407-10K]	5.3386932	1.087822	56	4.91	<.0001*
Time (day)[56]*System[P407-10K]	9.0603416	1.087822	56	8.33	<.0001*
Time (day)[63]*System[P407-10K]	4.8498776	1.087822	56	4.46	<.0001*

Table S4. Repeated measure analysis was conducted to assess the significant difference in the adalimumab cumulative release between P407-10K and P407-20K gels: In the model, time, system was taken into account to assess the system impact. Samples were incorporated as a random effect. Cumulative release of adalimumab between P407-10K and P407-20K is statistically significantly different from one another, except for days 1 to 7 and day 42. Prob>|t| denotes the p value for the two-tailed test. p <0.05 was considered significantly different from each other.

Response Cumulative human serum IgG release (%)

Effect Summary

Summary of Fit

RSquare	0.999815
RSquare Adj	0.999726
Root Mean Square Error	0.54731
Mean of Response	35.33425
Observations (or Sum Wgts)	90

Parameter Estimates

Term	Estimate	Std Error	DFDen	t Ratio	Prob>|t|
Intercept	35.33425	0.228273	4	154.79	<.0001*
Time (day)[0]	-35.33425	0.215862	56	-163.7	<.0001*
Time (day)[1]	-27.71863	0.215862	56	-128.4	<.0001*
Time (day)[2]	-25.05633	0.215862	56	-116.1	<.0001*
Time (day)[3]	-23.29321	0.215862	56	-107.9	<.0001*
Time (day)[4]	-21.63008	0.215862	56	-100.2	<.0001*
Time (day)[7]	-19.20592	0.215862	56	-88.97	<.0001*
Time (day)[9]	-17.50425	0.215862	56	-81.09	<.0001*
Time (day)[14]	-14.43279	0.215862	56	-66.86	<.0001*
Time (day)[21]	-9.4855	0.215862	56	-43.94	<.0001*
Time (day)[28]	-1.329458	0.215862	56	-6.16	<.0001*
Time (day)[35]	9.791375	0.215862	56	45.36	<.0001*
Time (day)[42]	20.509708	0.215862	56	95.01	<.0001*
Time (day)[49]	38.643458	0.215862	56	179.02	<.0001*
Time (day)[56]	59.720333	0.215862	56	276.66	<.0001*
System[P407-10K]	-3.351972	0.228273	4	-14.68	0.0001*
Time (day)[0]*System[P407-10K]	3.3519722	0.215862	56	15.53	<.0001*
Time (day)[1]*System[P407-10K]	0.3263472	0.215862	56	1.51	0.1362
Time (day)[2]*System[P407-10K]	-0.509278	0.215862	56	-2.36	0.0218*
Time (day)[3]*System[P407-10K]	-0.749903	0.215862	56	-3.47	0.0010*
Time (day)[4]*System[P407-10K]	-1.458861	0.215862	56	-6.76	<.0001*
Time (day)[7]*System[P407-10K]	-2.299694	0.215862	56	-10.65	<.0001*
Time (day)[9]*System[P407-10K]	-2.838028	0.215862	56	-13.15	<.0001*
Time (day)[14]*System[P407-10K]	-3.843236	0.215862	56	-17.80	<.0001*
Time (day)[21]*System[P407-10K]	-4.901361	0.215862	56	-22.71	<.0001*
Time (day)[28]*System[P407-10K]	-6.024903	0.215862	56	-27.91	<.0001*
Time (day)[35]*System[P407-10K]	-3.889903	0.215862	56	-18.02	<.0001*
Time (day)[42]*System[P407-10K]	-0.973653	0.215862	56	-4.51	<.0001*
Time (day)[49]*System[P407-10K]	7.1021806	0.215862	56	32.90	<.0001*
Time (day)[56]*System[P407-10K]	15.016139	0.215862	56	69.56	<.0001*

Table S5. Repeated measure analysis was conducted to assess the significant difference in the human serum IgG cumulative release between P407-10K and P407-20K gels: In the model, time, system was taken into account to assess the system impact. Samples were incorporated as a random effect. Cumulative release of human serum IgG between P407-10K and P407-20K is statistically significantly different from one another, except for days 1 and day 2. Prob>|t| denotes the *p* value for the two-tailed test. $p < 0.05$ was considered significantly different from each other.

Ordinary one-way ANOVA
Multiple comparisons

Number of families	1
Number of comparisons per family	6
p value threshold	0.05

**Tukey's multiple comparisons test**	Mean Diff.	95.00% CI of diff.	Adjusted *p* Value	Below threshold?	Result
Adalimumab -80°C vs. P407-10K Day 63	-29.9	-147.4 to 87.64	0.8461	No	ns
Adalimumab -80°C vs. P407-20K Day 70	-6.8	-124.3 to 110.7	0.9976	No	ns
Adalimumab -80°C vs. Adalimumab 37°C Day 70	-24.8	-142.3 to 92.74	0.9034	No	ns
P407-10K Day 63 vs. P407-20K Day 70	23.1	-94.44 to 140.6	0.9197	No	ns
P407-10K Day 63 vs. Adalimumab 37°C Day 70	5.1	-112.4 to 122.6	0.999	No	ns
P407-20K Day 70 vs. Adalimumab 37°C Day 70	-18	-135.5 to 99.54	0.9591	No	ns

Table S6. Ordinary one-way ANOVA analysis followed by Tukey's multiple comparison test was conducted to compare the IC50 values between the four groups (n=3). *p* value lower than 0.05 was considered to be statistically significant.

References

(1) Rubinstein, M.; Colby, R. Polym. Phys. In *Polym. Phys.*, 2003.
(2) Karvinen, J.; Ihalainen, T. O.; Calejo, M. T.; Jönkkäri, I.; Kellomäki, M. Characterization of the microstructure of hydrazone crosslinked polysaccharide-based hydrogels through rheological and diffusion studies. *Mater Sci Eng C Mater Biol Appl* **2019**, *94*, 1056-1066.
(3) Calvet, D.; Wong, J. Y.; Giasson, S. Rheological monitoring of polyacrylamide gelation: Importance of cross-link density and temperature. *Macromolecules* **2004**, *37* (20), 7762-7771.
